# Design principles for (efficient) excited-state absorption-based blue-to-UV upconversion phosphors with Pr^3+^†

**DOI:** 10.1039/d5sc01862e

**Published:** 2025-06-02

**Authors:** Tom Förster, Josefine Reifenberger, Tugce Moumin, Justus Helmbold, Željka Antić, Miroslav D. Dramićanin, Markus Suta

**Affiliations:** a Inorganic Photoactive Materials, Institute of Inorganic and Structural Chemistry, Heinrich Heine University Düsseldorf, Universitätsstraße 1 40225 Düsseldorf Germany markus.suta@hhu.de; b National Institute of Research and Development for Electrochemistry and Condensed Matter, INCEMC Timisoara Romania zeljkaa@gmail.com dramican@vinca.rs; c Centre of Excellence for Photoconversion, Vinča Institute of Nuclear Sciences, National Institute of the Republic of Serbia, University of Belgrade P. O. Box 522 11351 Belgrade Serbia

## Abstract

UV light generation is generally not very efficient, expensive, or may even require toxic elements such as mercury. In contrast, blue light (*λ* = 450 nm) is cheaply available from semiconductor LEDs and its use in phosphor-converted LEDs is technologically mature and could be envisioned as an intense, sustainable light source in an upconversion scheme. The electronic energy level landscape of the 4f^2^ ion Pr^3+^ does allow such a blue-to-UV upconversion (UC) by resonantly exciting the ^3^P_*J*_ (*J* = 0, 1, 2) levels with blue light, followed by absorption of a second blue photon, thus populating the 4f^1^5d^1^ configuration states located in the UV range. While the second absorption step is expected to be efficient based on selection rules, no clear guidelines on how to optimize the expected upconversion efficiency for Pr^3+^ by appropriate choice of a surrounding host are known up to now. Within this work, selected halidoelpasolites, oxyfluorides, garnets, silicates and borates are activated with Pr^3+^ to understand the relation between ESA-based UC efficiency, the energy and configurational offset of the 4f^1^5d^1^ states as well as the excited-state dynamics. For that purpose, quantum yield measurements, as well as steady-state, time-resolved and temperature-dependent luminescence spectroscopy with different excitation sources and powers are combined. It turns out that several parameters must be carefully mutually matched within a host compound for efficient ESA-based blue-to-UV UC with Pr^3+^. Not only does the decay time of the intermediate ^3^P_0_ level have to be particularly long in an excited-state absorption upconversion scheme, but also the non-radiative crossover from the excited 4f^1^5d^1^ states needs to be limited. All these conditions are particularly well fulfilled in the Pr^3+^-activated chloridoelpasolite Cs_2_NaYCl_6_:Pr^3+^, which shows the highest upconversion quantum yield (*Φ*_UC_ = 0.11%, *P* = 0.59 W cm^−2^) among all investigated compounds within this work and even surpasses the efficiency of well-known upconverters in this field such as Lu_3_Al_5_O_12_:Pr^3+^ (LuAG:Pr^3+^) or β-Y_2_Si_2_O_7_:Pr^3+^ (YPS:Pr^3+^). The relatively high efficiency of this compound compared to the other standards is a consequence of its low cut-off phonon energy and rigid, densely packed structure with large mutual distances between the rare-earth ions.

## Abstract 2

Guidelines for efficient blue-to-UV upconversion with the lanthanoid ion Pr^3+^ are established and experimentally tested. It turns out that Cs_2_NaYCl_6_:Pr^3+^ is an interesting candidate for efficient upconversion by this proposed scheme.

## Graphical abstract

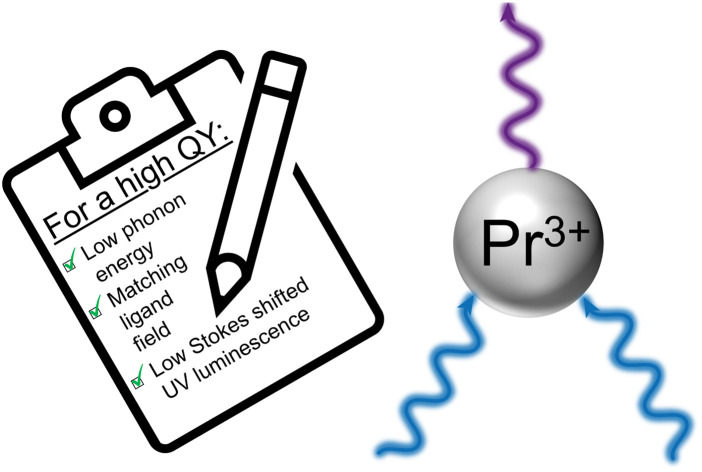

## 1 Introduction

The generation of ultraviolet (UV) light is essential for the fields of photocatalysis,^1,2^ antimicrobial treatments,^3–5^ cancer therapy^6^ and persistent optical tags.^7^ Historically, UV light is directly generated from sealed mercury (253.7 nm), xenon (190–1100 nm), or deuterium (190–370 nm) gas-based light sources. The current industrial standard for UVC light sources is the electric discharge of mercury, which has many disadvantages such as short lifetimes, large heat generation, and safety hazards. In addition to these, excimer lasers are used as high-energy UV light sources.^8^ These are also characterised by high voltages and safety hazards due to handling with poisonous gases (halogens).^9^ Due to their more complex set-ups, mercury lamps and excimer lasers are very limited in size.

Another modern possibility would be the use of UV light emitting diodes (UV-LEDs), which however show a strong drop in their external quantum efficiencies for emission wavelengths below 365 nm and potent doped semiconductors are not always readily available.^10–13^

An alternative approach is the generation of UV light through blue-to-UV upconversion (UC).^7,14^ Despite a theoretical maximum quantum yield of 50% (ref. 15) due to the absorption of two photons, there is the cost-effective and technologically established possibility of applying a powdered phosphor to a blue light LED chip analogous to a phosphor-converted LED (pc-LED)^16^ to generate UV light.

By absorbing two or more long-wavelength photons, both organic and inorganic compounds can convert these into a UV light photon in an anti-Stokes process.^17^ For organic compounds, usually triplet–triplet annihilation is the working mechanism, giving rise to decently efficient blue-to-UV upconversion (UC efficiency *η*_UC_ ∼20%, UC quantum yield *Φ*_UC_ ≤ 5.7%).^18–20^ In inorganic materials, mostly lanthanide-activated host compounds have emerged as UV upconversion systems, especially Yb^3+^/Tm^3+^ co-activated materials for a NIR-to-UV upconversion process.^21–23^ However, this is a 5-photon process, which results in an overall low efficiency for the generation of UV light (*Φ*_UC_(250–375 nm) ≈10^−5^ for LiYF_4_: 0.4 mol% Tm^3+^, 16.5 mol% Yb^3+^ single crystal at 10 W cm^−2^ (ref. 24)).

**Fig. 1:**
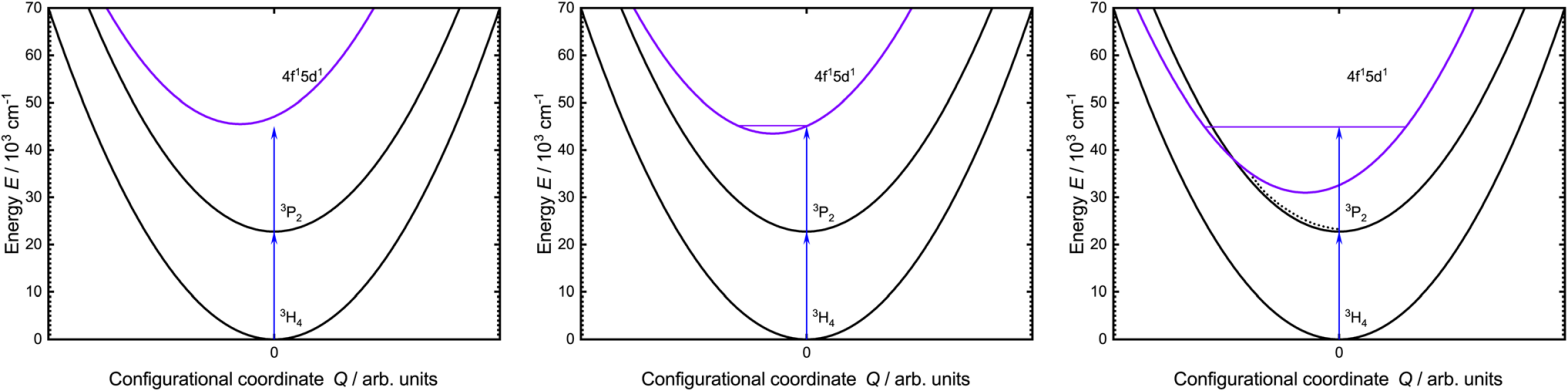
Simplified configurational coordinate diagram of Pr^3+^ showing the involved states of the blue-to-UV upconversion process. (Left) 4f^1^5d^1^ states are not accessible *via* blue-to-UV upconversion. (Middle) 4f^1^5d^1^ states are in the right range for blue-to-UV upconversion. (Right) Blue-to-UV upconversion results in an excitation above the crossover of the 4f^1^5d^1^ states, which results in non-radiative relaxation into the lower-lying 4f^2^ levels (dotted line).

In the last few years, Pr^3+^-activated ([Xe]4f^2^) materials have become increasingly interesting for blue-to-UV upconversion.^25–29^ The electronic structure of Pr^3+^ allows upconversion to the states of the excited [Xe]4f^1^5d^1^ configuration upon irradiation with intense blue light. Two basic mechanisms are particularly relevant for upconversion, which are majorly dependent on the activator concentration. For low concentrations, two sequential one-photon absorption processes give rise to collective single-ion upconversion based on excited-state absorption (ESA). This mechanism is typically rather inefficient.^17^ At higher activator fractions, it is possible that two neighboring ions can be excited and upconversion can occur by an energy transfer process.^30^ This process is referred to as energy transfer upconversion (ETU) and typically more efficient than single ion-based ESA. For Pr^3+^, intense blue excitation into the ^3^P_*J*_ levels (*J* = 0, 1, 2) results in a fast decaying (∼10 ns) broadband UV emission due to an electric-dipole allowed 4f^1^5d^1^ → 4f^2^ transition.^25,31–33^ This type of electric-dipole allowed transitions is widely known from other lanthanides, such as Ce^3+^ (ref. 34–36) and divalent lanthanides like Eu^2+^ (ref. 37 and 38), Yb^2+^ (ref. 39 and 40) or Sm^2+^ (ref. 41 and 42). Due to the involvement of the 5d orbitals and the resulting crystal field splitting, the energy of the excited 4f^1^5d^1^ states can be tuned by the chemical composition, Pr–ligand distance, and local site symmetry of the Pr^3+^ ions.^43,44^ Precise tuning of the energy position of the 4f^1^5d^1^ configuration states is mandatory: if the energy of the 4f^1^5d^1^ states is too high (Δ*E*(^3^H_4_, 4f^1^5d^1^) > 44 445 cm^−1^), two blue photons will not lead to a resonant upconversion process. If the energy of the 4f^1^5d^1^ states is, however, too low, the excess energy of the two blue photons may provoke a non-radiative crossover or even thermal ionization into the conduction band ultimately quenching the 4f^1^5d^1^ → 4f^2^-based emission (see Fig. 1).^45–47^

A very well-established upconversion phosphor is β-NaYF_4_:Er^3+^,Yb^3+^, in which the Yb^3+^ efficiently absorb NIR light resulting in upconverted green and red emission by the Er^3+^ ions.^48–52^ The Yb^3+^/Er^3+^ NIR-to-Vis UC follows the cooperative ETU mechanism^53^ and is quite efficient, as upconversion quantum yields up to 10% can be achieved in β-NaYF_4_: 2% Er^3+^, 18% Yb^3+^ by now.^54–56^ This is the result of decades of research into optimising this cooperative ETU pair.^57,58^ However, for Pr^3+^, UC quantum yields are much lower (*Φ*_UC_ < 1%),^59^ as there are no guidelines for designing Pr^3+^-based upconversion phosphors yet, although many examples are already known in the literature.^29,47,60–65^ Unfortunately, the well-known host compounds for upconversion, α-/β-NaYF_4_ and LiYF_4_ are not suitable for the blue-to-UV upconversion of Pr^3+^. In the case of α-NaYF_4_, the 4f^1^5d^1^ states are energetically above the ^1^S_0_ (4f^2^) level, resulting in quantum cascade luminescence.^66^ For β-NaYF_4_ and LiYF_4_, on the other hand, the excitation of the 4f^1^5d^1^ states is outside the energy range (>42 700 cm^−1^).^67–69^

**Fig. 2:**
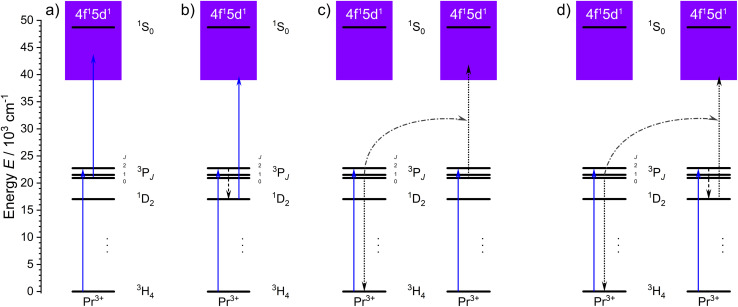
Possible blue-to-UV upconversion mechanisms of Pr^3+^ after first excitation with blue light populating the ^3^P_2_ level. Solid arrows represent absorption, while dotted arrows show non-radiative processes. (a) Excited-state absorption involving ^3^P_0_ as intermediate state. (b) Excited-state absorption involving ^1^D_2_ as intermediate state. (c) Energy transfer upconversion involving ^3^P_0_ as intermediate state. (d) Energy transfer upconversion involving ^1^D_2_ as intermediate state.

A special feature of the electronic energy level landscape of Pr^3+^ is that both ESA and ETU are in principle possible as UC mechanisms (Fig. 2). ETU can be controlled *via* the activator concentration in a given host compound. Since ETU is more efficient than ESA, a high concentration of Pr^3+^ may appear as an obvious choice. However, both the excited ^3^P_0_ and ^1^D_2_ levels of Pr^3+^ also show a high tendency of cross-relaxation at elevated activator concentrations, which increases their non-radiative decay rates.^70–72^ This results in an additional difficulty for ETU-based blue-to-UV UC with Pr^3+^. Consequently, the ESA process should first be optimised at low concentrations of Pr^3+^ for a better understanding of the control of the upconversion process in general.

A long-lived intermediate state is required for efficient ESA. The decay rate of the ^3^P_0_ level is thus very crucial for Pr^3+^, which is mainly controlled by multiphonon relaxation (MPR) at low concentrations.^73^ According to Hund's rule, the spin multiplicity of the lowest 4f^1^5d^1^ state should be dominantly triplets given the still limited degree of spin–orbit coupling. Consequently, ESA from the intermediate ^3^P_0_ level is expected to be more effective than from the ^1^D_2_ level.^60,74,75^ There have been recent reports about a potentially important role of the lower energetic ^1^D_2_ level in the blue-to-UV upconversion with Pr^3+^ in the case of Sr_3_(BO_3_)_2_:Pr^3+^ (ref. 61). Within this work, however, we will explicitly focus on the ESA-based upconversion mechanism and the importance of the ^3^P_0_ level, while a direct inclusion of the ^1^D_2_ level will be part of future studies.

The efficiency of ESA-based blue-to-UV upconversion in Pr^3+^-activated phosphors is critically affected by the decay rate of the ^3^P_0_ state and the crossover energy of the 4f^1^5d^1^ states. To prove this, we systematically elucidated different microcrystalline phosphors activated with 0.5 mol% Pr^3+^, namely YAl_3_(BO_3_)_4_ (YAB),^28^ Na_3_Y(BO_3_)_2_ (NYB),^76^ β-Y_2_Si_2_O_7_ (YPS),^75,77^ X2-Y_2_SiO_5_ (YSO),^78–80^ Lu_3_Al_5_O_12_ (LuAG),^81,82^ Y_3_Al_5_O_12_ (YAG),^82^ Y_7_O_6_F_9_ (V-YOF)^27^ and Cs_2_NaYCl_6_.^83^ These host compounds differ in their cut-off phonon energies, but also have small sites and matching energies of the 4f^1^5d^1^ configuration states. The host compounds are activated with a low concentration of Pr^3+^ to prevent cross-relaxation and ETU. It is important to note that the optimum Pr^3+^ concentration also depends on the explicit structure of the surrounding host compounds, which has an immediate impact on the achievable (internal) quantum yield.

## 2 Results and discussion

### 2.1 Structural analysis

The X-ray powder diffraction (XRPD) patterns of the synthesized microcrystalline powders are depicted in Fig. S1,† showing no additional diffraction peaks and therefore no detectable impurities for most of the powders. Small traces of α-cristobalite (SiO_2_, ICSD depository no.: 77 452 (ref. 84)) are detected in the XRPD pattern of YPS due to the employed excess of TEOS in the synthesising process. Furthermore, other Bragg reflections can be observed in LuAG, which can be assigned to the used aluminum sample holder. Based on Rietveld refinement, the obtained lattice parameters were slightly larger than in the pure compounds (Tables S1–S8†). This observation can be attributed to the higher ionic radius of Pr^3+^ compared to Y^3+^ and Lu^3+^.^85^

### 2.2 Photophysical properties

#### 2.2.1 Role of the intermediate ^3^P_0_ level

Fig. S4† depicts the excitation spectra of the obtained samples at room temperature. It contains narrow excitation bands around 450 nm related to the ^3^P_0, 1, 2_, ^1^I_6_ ← ^3^H_4_ transitions of the incorporated Pr^3+^ ions, in good agreement with earlier reports.^86–89^

**Fig. 3:**
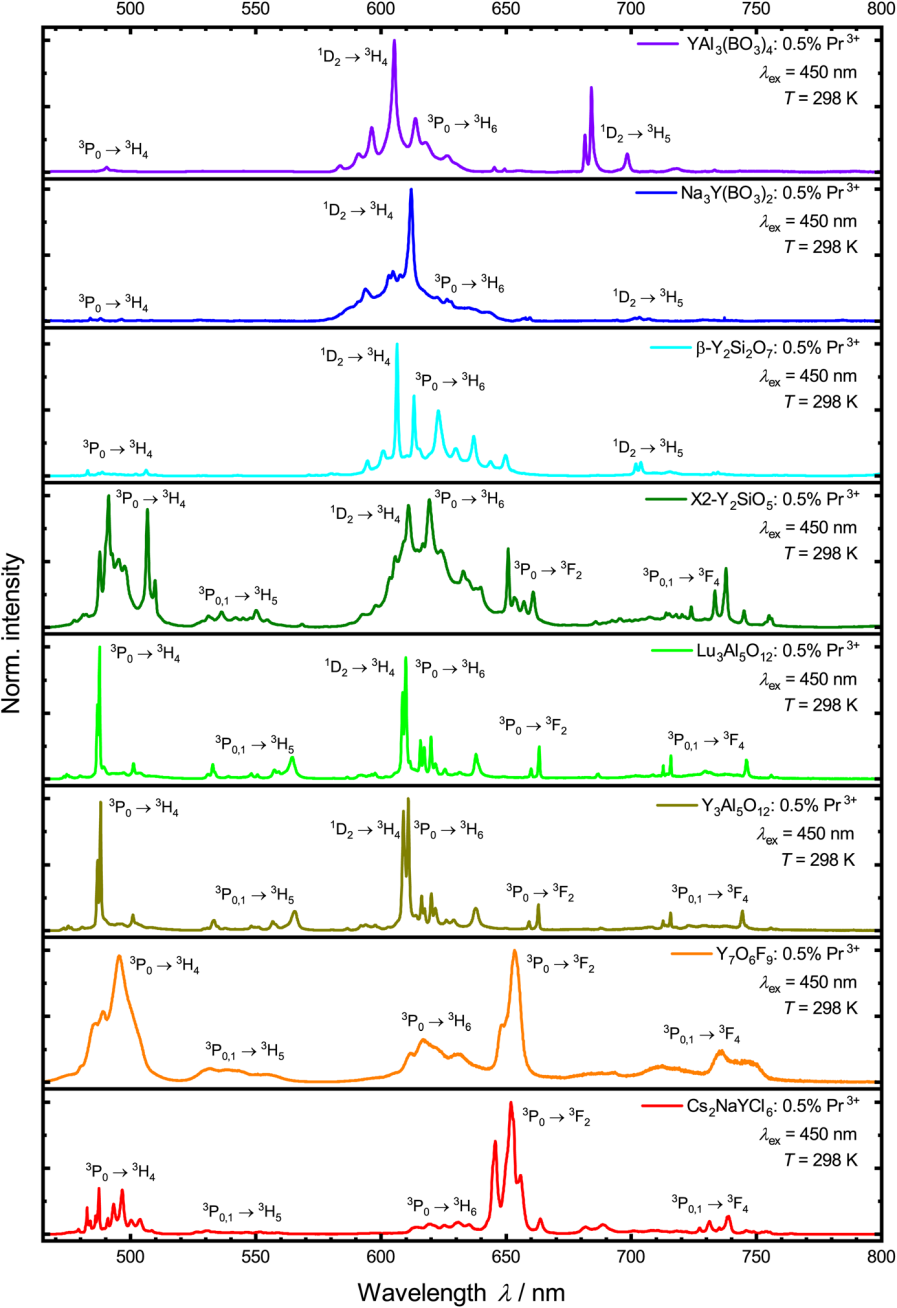
Normalized photoluminescence emission spectra of the synthesized samples under 450 nm excitation into the ^3^P_2_ level at 298 K.

**Fig. 4:**
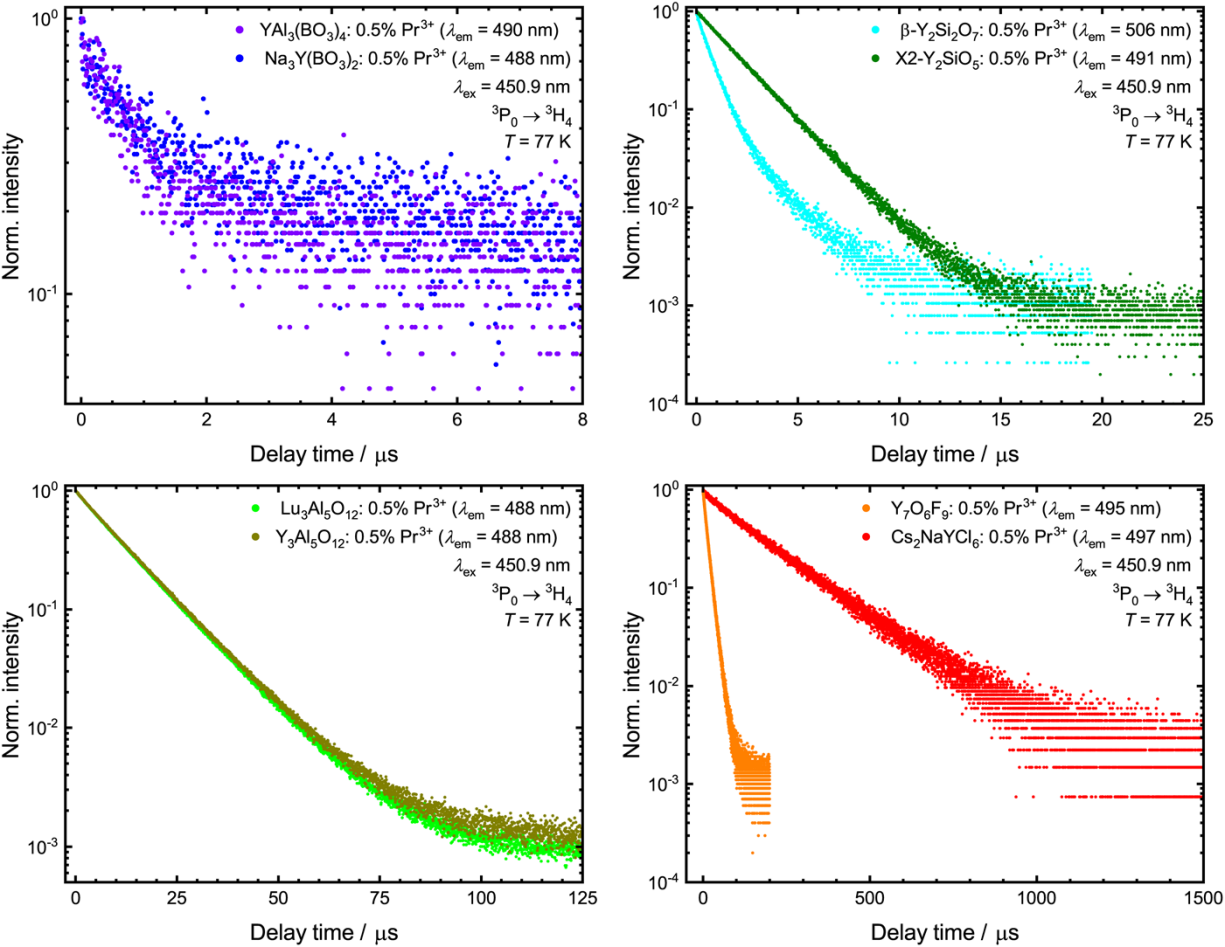
Photoluminescence decay curves of the synthesized samples of the ^3^P_0_ level under 450.9 nm excitation (^3^P_2_ ← ^3^H_4_) at 77 K monitoring the ^3^P_0_ → ^3^H_4_-based emission around 488 nm. Due to strongly differing decay times, decay curves are shown for different delay times. Values of the measured decay times are given in Table 1.

All the Pr^3+^-activated samples show luminescence in the visible range under blue light excitation into the ^3^P_2_ level (Fig. 3). There are up to nine sets of emission bands in the spectra, displaying several transitions from the ^3^P_1_, ^3^P_0_ and ^1^D_2_ levels into the energetically lower ^3^H_*J*_ (*J* = 4, 5, 6) and ^3^F_*J*′_ (*J*′ = 2, 3, 4) levels. The Pr^3+^ 4f^2^–4f^2^-based emission in V-YOF is comparably broadened, which can be attributed to the presence of four crystallographically independent Y^3+^ sites in the structure.^32^ The most evident difference among the luminescence spectra of the various presented Pr^3+^-activated compounds is the intensity of the ^1^D_2_ → ^3^H_4_ transition: in hosts with a high cut-off phonon energy, *e.g.* borates and silicates (exact values of cut-off phonon energies are compiled in Table 1, as derived from the IR spectra shown in the ESI†), the ^1^D_2_ → ^3^H_4_ transition is the most intense transition in the spectrum, while in hosts with comparably low cut-off phonon energies, it is almost absent in favor of ^3^P_0_-based luminescence. That striking difference is in line with the energy gap law of multiphonon transitions, which states that the non-radiative decay rate is exponentially damped with increasing number of required phonons to bridge the energy gap between two energetically neighboring energy levels. The energy gap Δ*E* between the ^3^P_0_ and ^1^D_2_ levels of Pr^3+^ is about 3700 cm^−1^.^90^ Therefore, in host compounds with high cut-off phonon energies such as borates (see Table 1 and Fig. S8–S15†), the ^3^P_0_ level quickly decays non-radiatively to the lower energetic ^1^D_2_ level, while in hosts with a comparably low cut-off phonon energy such as a chloride, non-radiative decay is slow compared to radiative decay resulting in a longer decay time of the ^3^P_0_ level (see Fig. 4 and Table 1). Another possibility for non-radiative relaxation from the ^3^P_0_ to the ^1^D_2_ level are intervalence charge transfer (IVCT) states between Pr^3+^ and transition metal ions with a d^0^ valence electron configuration such as Ti^4+^, V^5+^, Zr^4+^ or Nb^5+^.^91–93^ In the host compounds considered within this work, however, IVCT states are not observable and a potential impact can be disregarded.

**Table 1:** Determined decay time for the ^3^P_0_ level *τ*(^3^P_0_), ^1^D_2_ level *τ*(^1^D_2_) and cut-off phonon energy *ℏω*_eff_ of the different compounds (see Section 4 and Fig. S5 in the ESI)

	*τ*(^3^P_0_) (77 K)/μs	*τ*(^1^D_2_) (298 K)/μs	*ℏω*_eff_/cm^−1^
YAB:Pr^3+^	0.85 ± 0.03	15.52 ± 0.04	1400
NYB:Pr^3+^	0.86 ± 0.04	13.99 ± 0.03	1395
YPS:Pr^3+^	1.03 ± 0.01	156.19 ± 1.09	1113
YSO:Pr^3+^	1.96 ± 0.01	71.35 ± 1.32	990
LuAG:Pr^3+^	11.75 ± 0.39	150.84 ± 0.92	850
YAG:Pr^3+^	12.01 ± 0.66	153.48 ± 1.43	840
V-YOF:Pr^3+^	13.48 ± 0.03	66.35 ± 1.21	520
Cs_2_NaYCl_6_:Pr^3+^	172.67 ± 0.29	1107.96 ± 7.68	287

The decay curves of the ^1^D_2_-based luminescence under direct excitation were also measured (see Fig. S5† and Table 1). In line with these observations, also the ^1^D_2_ level follows this general trend, although its decay time is generally longer than that of the ^3^P_0_ level based on its energetically more isolated nature (Δ*E* = 7000 cm^−1^ to the lower ^1^G_4_ level^90^).

#### 2.2.2 4f^1^5d^1^ → 4f^2^ broadband emission

**Fig. 5:**
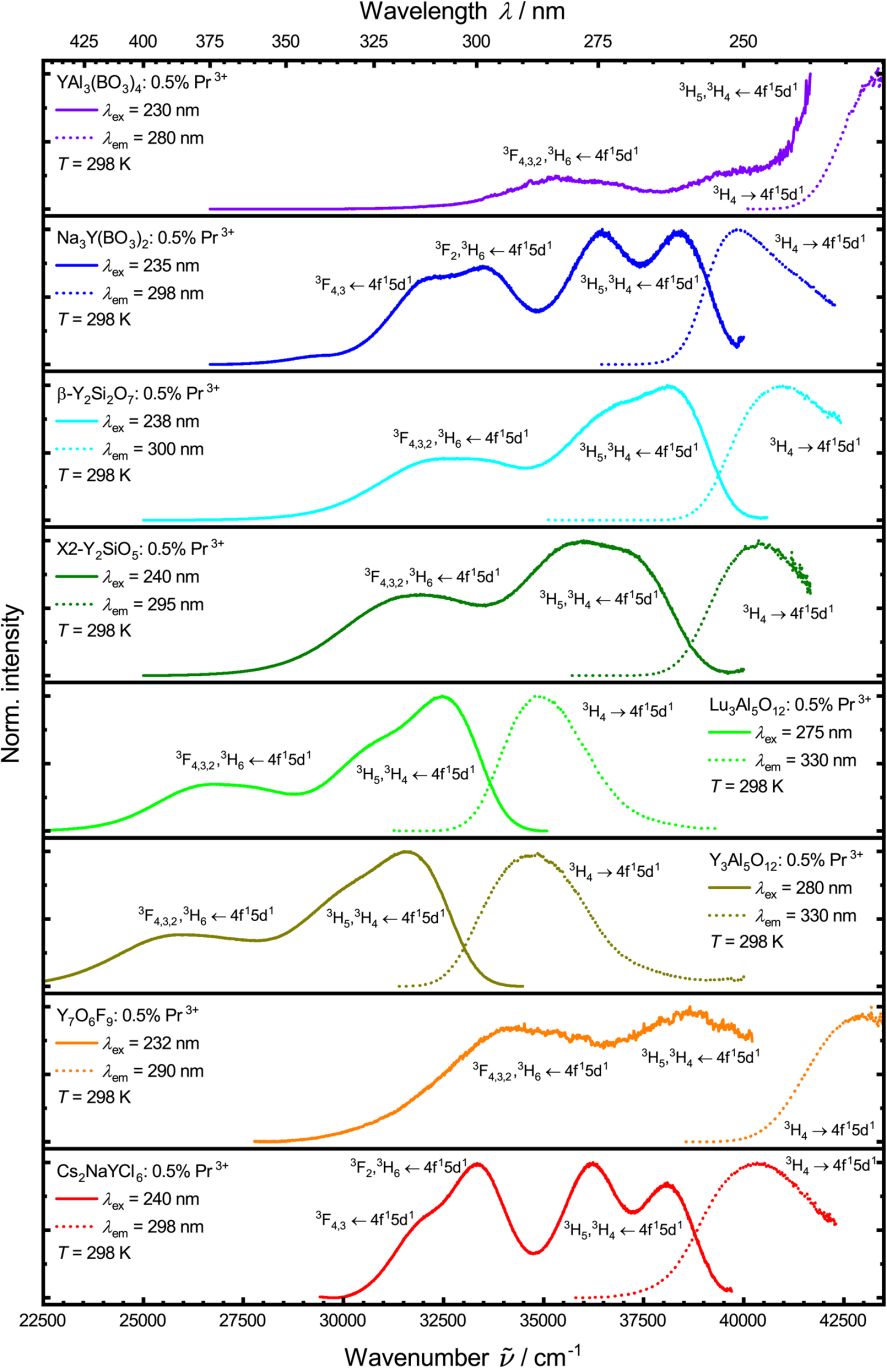
Normalized photoluminescence emission (solid lines) and excitation spectra (dotted lines) of the synthesized samples at 298 K in UV range showing emission and excitation of 4f^1^5d^1^ states. Spectra were measured against wavelengths and converted to wavenumbers using a Jacobian transformation.^99^

Excitation with UV light (*λ*_ex_ = 250–300 nm) results in broad banded UV emission of Pr^3+^ from the excited 4f^1^5d^1^ configuration (see Fig. 5). Due to lacking transitions from the excited ^1^S_0_ (4f^2^) level, it can be concluded that the lowest 4f^1^5d^1^ state is energetically below this state, as known from other host matrices.^94,95^ Given the electric-dipole allowed character of the 4f^1^5d^1^ → 4f^2^ transition, the decay time is in the range of 10–30 ns (ref. 96–98) (see Fig. S7†).

The variation of the 4f^1^5d^1^-related decay times among the various host compounds has several reasons. On the one hand, the decay time depends on the thermal activation barrier for non-radiative crossover to the lower energy 4f^2^ (^3^P_*J*_, ^1^D_2_) states.^46,47,100,101^ Photoluminescence excitation spectra monitoring the respective ^3^P_0_- or ^1^D_2_-based emission can give additional insights here as a broad excitation band in the UV range indicates indirect excitation *via* the 4f^1^5d^1^ states by non-radiative crossover. The other influences are the wavelength of the 4f^1^5d^1^ → 4f^2^-based emission and the refractive index of the host compound at that given wavelength, which affects the available photonic density of states and thus, the radiative decay probability itself.^102,103^

From the 4f^1^5d^1^ ↔ 4f^2^-based broadband emission and excitation spectra of Pr^3+^ in the UV range at 77 K (Fig. S6†), Stokes shifts can be estimated that give additional insights into the degree of non-radiative relaxation by thermal crossover. They are compiled in Table 2. For that, emission spectra recorded in wavelengths were converted to wavenumbers using a Jacobian transformation.^99^ Emission and excitation spectra were fitted using Gaussian fits, calculating the Stokes shift between the lowest energy (excitation) and the highest energy (emission) maximum.

**Table 2:** Determined Stokes shift Δ*S* (Fig. S6) and decay times for the 4f^1^5d^1^ configuration states *τ*(4f^1^5d^1^) of the Pr^3+^-based luminescence at 77 K

	Δ*S* (77 K)/cm^−1^	*τ*(4f^1^5d^1^) (77 K)/ns
YAB:Pr^3+^	—	—
NYB:Pr^3+^	1390	14.00 ± 0.12
YPS:Pr^3+^	2010	19.35 ± 0.13
YSO:Pr^3+^	2530	19.13 ± 0.05
LuAG:Pr^3+^	1970	18.95 ± 0.01
YAG:Pr^3+^	2550	23.57 ± 0.02
V-YOF:Pr^3+^	3150^a^	—
Cs_2_NaYCl_6_:Pr^3+^	1520	16.93 ± 0.06

a Value according to spectra at 298 K.

The Stokes shift of the Pr^3+^-activated YAB was not determined due to limitations in the experimental setup. From the depicted spectra at 298 K (Fig. 5), it can be inferred, however, that the 4f^1^5d^1^ → 4f^2^-based emission in YAB:Pr^3+^ has a small Stokes shift. Pr^3+^ shows the lowest Stokes shift in NYB (1390 cm^−1^), which is in good agreement with Pr^3+^ in other borates, like YBO_3_ (1800 cm^−1^).^104^ The determined Stokes shift in Cs_2_NaYCl_6_ (1520 cm^−1^) is in good agreement with previous results (1028 cm^−1^ (ref. 105) at 10 K). The Stokes shifts of the respective emission of Pr^3+^ in YAG (2550 cm^−1^) and LuAG (1970 cm^−1^) are also in good agreement with previous reports.^46,95,106^ The lower Stokes shift in LuAG compared to YAG results from the smaller rare earth site the Pr^3+^ ions occupy.^104^ Previously reported values of the Stokes shift of Pr^3+^ in YSO are in a similar range (≈2420 cm^−1^ (ref. 106); ≈ 2852 cm^−1^ (ref. 74), calculated from emission and excitation maxima) to the one determined here (2530 cm^−1^). For Pr^3+^ in YPS, our determined Stokes shift (2010 cm^−1^) fits well to those estimated by other groups (≈2265 cm^−1^ (ref. 75), calculated from emission and excitation maxima).

Due to limitations in the experimental setup, a Stokes shift of Pr^3+^-activated V-YOF could not be determined from spectra at 77 K. Therefore, the Stokes shift was taken from spectra at 298 K for the sake of comparison. Pr^3+^-activated V-YOF shows the highest Stokes shift of the investigated materials (3150 cm^−1^). This value is in agreement with previously published results (≈3499 cm^−1^ (ref. 27), calculated from emission and excitation maxima). As there is weak, but still observable UV emission from Pr^3+^ in V-YOF, non-radiative relaxation from 4f^1^5d^1^ → 4f^2^ and 4f^1^5d^1^ radiative decay starts to compete. This competition is particularly severe at room temperature with a connected Stokes shift of around 3000 cm^−1^.^107,108^

**Fig. 6:**
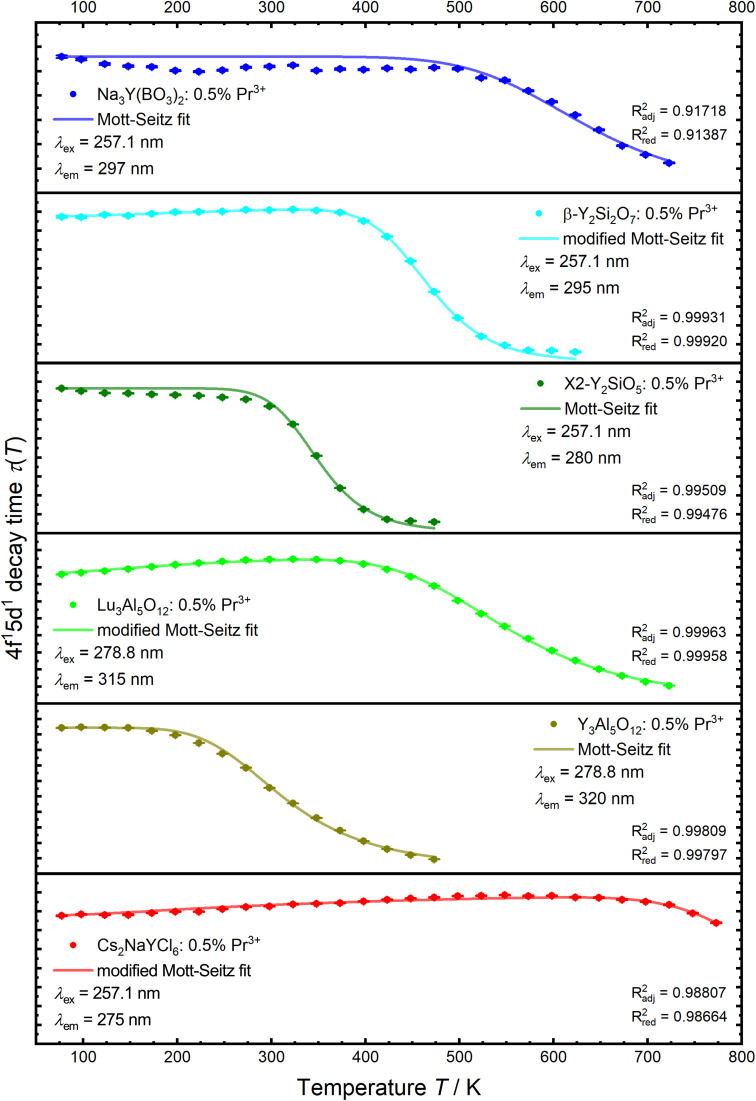
Luminescence decay times of 4f^1^5d^1^ configuration states of Pr^3+^ in the synthesized host compounds as a function of temperature with a fit to a Mott-Seitz law (eqn (1) or (3)). Decay times were derived from the exponential fit using eqn (S1).†

Due to the involvement of the spatially more extended 5d orbitals in the excited 4f^1^5d^1^ configuration, a shift in the configuration coordinate diagram is to be expected. This leads to a crossover with lower lying 4f^2^ levels, *via* which the 4f^1^5d^1^ state can be non-radiatively depopulated upon thermal activation. It is already known that the position of the crossover barrier correlates with the Stokes shift.^45,46^ The crossover barrier is also relevant for the thermal quenching of the 4f^1^5d^1^ → 4f^2^-based broadband emission and can be probed by the temperature-dependent decay times of the 4f^1^5d^1^ states (Fig. 6).

The temperature dependence follows a Mott-Seitz law^109^ (eqn (1)) and allows the estimation of the crossover barrier and thermal quenching temperature *T*_50_ (eqn (2)) defined as the temperature at which the decay time has decreased to 50% of its original value at sufficiently low temperatures (77 K). The quenching temperatures for the different regarded Pr^3+^-activated compounds are compiled in Table 3.1
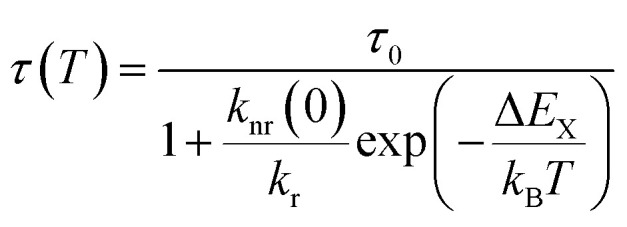
2
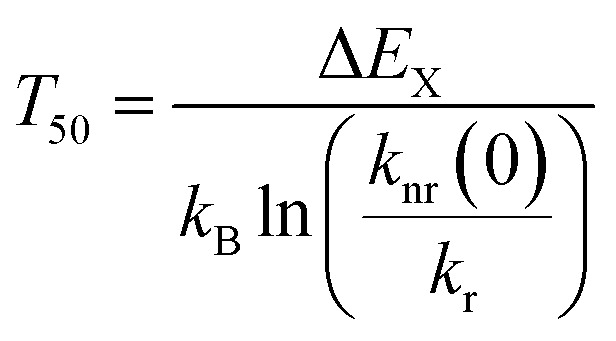


**Table 3:** Obtained quenching temperature *T*_50_ of the 4f^1^5d^1^ decay time of Pr^3+^ in the activated hosts. For Pr^3+^-activated YAB and V-YOF, no measurements could be done due to limitations in the experimental setup. Due to the high Stokes shift of Pr^3+^ in V-YOF (Table 2), a lower *T*_50_ than YAG:Pr^3+^ is assumed

	*T*_50_/K
YAB:Pr^3+^	—
NYB:Pr^3+^	∼634
YPS:Pr^3+^	∼467
YSO:Pr^3+^	∼350
LuAG:Pr^3+^	∼552
YAG:Pr^3+^	∼318
V-YOF:Pr^3+^	<318
Cs_2_NaYCl_6_:Pr^3+^	∼845

For YPS:Pr^3+^, LuAG:Pr^3+^ and Cs_2_NaYCl_6_:Pr^3+^ an increase of 4f^1^5d^1^-related decay time can be observed with increasing temperature before quenching. This behaviour is also known for the electric-dipole allowed 4f^6^5d^1^ → 4f^7^ transition of Eu^2+^ due to mixing of spin-forbidden components into the spin-allowed component.^110–112^ This could also happen for Pr^3+^ as the 4f^1^5d^1^ states can have spin triplet and singlet character.^113,114^ Consequently, the regular Mott-Seitz law can no longer be fully applied for this compounds. Therefore, the Mott-Seitz law is extended by a Boltzmann expression for *τ*_0_ (ref. 115) in eqn (1), which takes into account a thermal coupling between the singlet and triplet states of the excited 4f^1^5d^1^ configuration of Pr^3+^. The added Boltzmann expression takes into account an effective energy gap between coupled singlet and triplet states Δ*E*_ST_, the decay rate of the singlet state *k*_r,S_ and the degeneracy *g*_1_ for the lower excited state |1〉 and the degeneracy *g*_2_ for the higher excited state |2〉 (*g* = 3 for triplet and *g* = 1 for singlet states), respectively,3
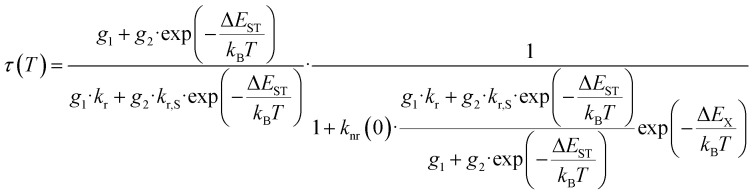


The lowest quenching temperature was determined for YAG:Pr^3+^ at ∼318 K, which is in good agreement with previous results.^46,82,106^ Quenching of the 4f^1^5d^1^ → 4f^2^-based luminescence of Pr^3+^ in YAG starts to become noticeable even at 150 K. This fundamental issue will also lower the overall expected upconversion quantum yield of this luminescent compound as the 4f^1^5d^1^ state of Pr^3+^ in YAG shows a strong tendency for thermally activated non-radiative decay even below room temperature. Quenching at room temperature is also a problem for YSO:Pr^3+^ with *T*_50_ ∼ 350 K for the 4f^1^5d^1^ → 4f^2^-based broadband emission. This value is in line with previous work.^100,106^ The other regarded Pr^3+^-activated samples were characterized by significantly higher quenching temperatures of the 4f^1^5d^1^ → 4f^2^-based luminescence.

Quenching temperatures of the samples are in good agreement with the determined Stokes shifts as a high Stokes shift generally scales with a low quenching temperature. Based on that argument, a very low *T*_50_ can be assumed for V-YOF:Pr^3+^, probably even lower than YAG:Pr^3+^.

#### 2.2.3 Upconversion luminescence

**Fig. 7:**
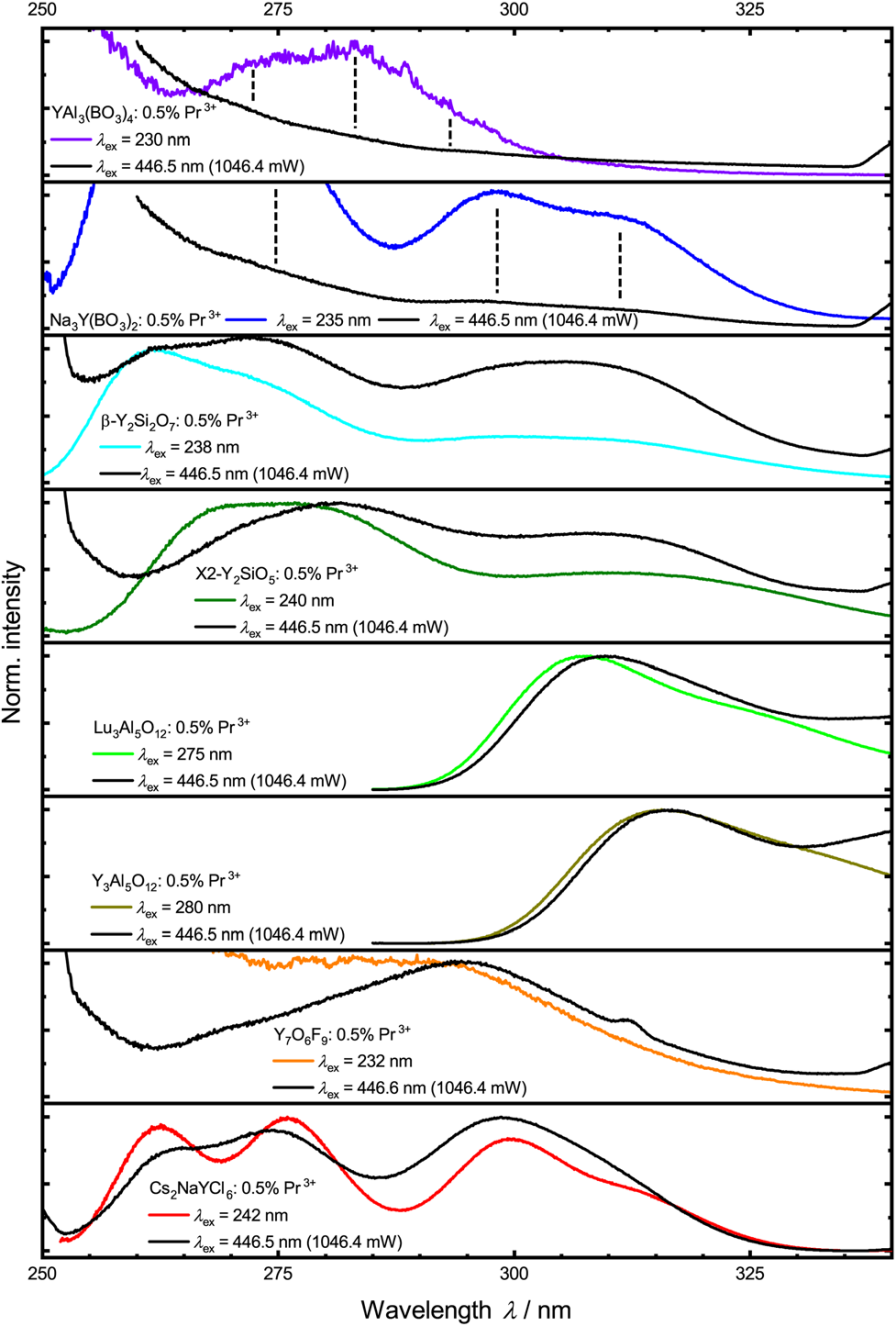
Comparison of UV emission under direct excitation (colored lines) and high power blue light excitation (black lines) for the different samples. Emission under blue light was only corrected for PMT sensitivity. Because of measurement artefacts, spectra are only shown up to 340 nm.

For UC luminescence, the investigated Pr^3+^-activated samples were irradiated with blue laser light of varying pump power (see the ESI† for spectra and pump power). A comparison of the resulting luminescence with luminescence under direct UV excitation clearly demonstrates that upconversion into the 4f^1^5d^1^ states upon excitation with blue light does work in the selected compounds (Fig. 7). Small shifts and changes in relative intensities can be attributed to the fact that instrument effects of the excitation source were not corrected for the upconversion spectra.

The number of photons *n* involved in the upconversion process can be estimated from the slope of a double logarithmic plot of the integrated upconversion intensity *I* against the incident pump power *P* since^116^

4 *I* ∝ *P*^*n*^

**Fig. 8:**
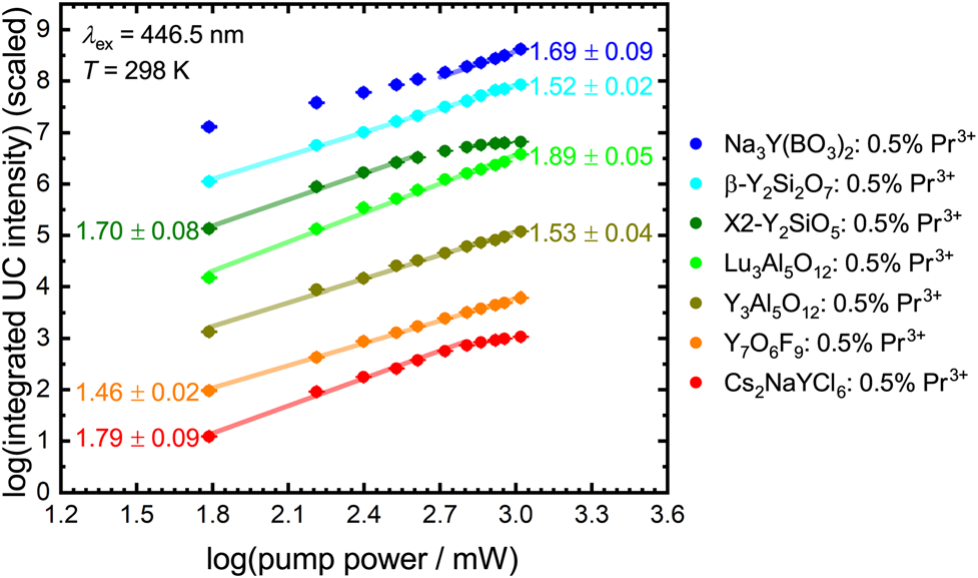
Double logarithmic plot of integrated intensity of the upconverted 4f^1^5d^1^-based emission of Pr^3+^ against blue laser (*λ*_ex_ = 446.5 nm) pump power at 298 K with linear regression. UC intensity is scaled to allow a better comparison. Slopes are given in the diagram.

Fig. 8 depicts power dependence for the investigated Pr^3+^-activated compounds within this work. The slope implies a two-photon process for most of the samples, in agreement with expectations. The low upconversion intensity of Pr^3+^ in YAB does not allow the determination of a reliable number of involved photons in this upconversion process as the intensity of the upconverted 4f^1^5d^1^-based luminescence is barely distinguishable from background noise and scattering light (Fig. S16†). The same observation can be made for NYB: 0.5 mol% Pr^3+^ at lower pump power. Previous research obtained a slope near two using Gd^3+^ as a sensitizer,^28^ so a two-photon process for YAB can also be assumed. Due to additionally active non-radiative processes in many of the investigated Pr^3+^-activated compounds at room temperature, the resulting linear fits give slopes lower than the value of 2.^116^ At higher incident pump power, YSO and Cs_2_NaYCl_6_ show saturation effects, which indicate that the intermediate level shows a small decay compared to the pump rate (see the ESI†).

Due to the low Pr^3+^ concentration, we assume ESA as a primary UC mechanism. However, to fully differentiate between ESA and ETU as a UC mechanism, kinetic rate equations according to Pollnau *et al.*^116^ and Sun *et al.*^78^ were used to describe the behaviour in the limits of low and high pump rates compared to the decay rate of the intermediate level. A complete derivation of the power-dependent excited-state kinetics is detailed in the ESI.†

It becomes evident that the UC mechanism of Pr^3+^-activated compounds cannot be determined *via* the intensity of the 4f^1^5d^1^ configuration states, as this has the same dependence on the irradiated power for both ESA and ETU (Table 4). However, the intensities of the ^3^P_0_ and the ^1^D_2_ level show a change in the power dependence with the onset of saturation. As depicted in Fig. 8, only Pr^3+^-activated YSO and Cs_2_NaYCl_6_ show saturation effects with our simple experimental setup. For YSO activated with 0.5 mol% Pr^3+^, ETU is assumed to be the dominant UC mechanism based on estimates,^78^ which can be explained by the presence of two different Y sites with a mutual distance of only 3.51 Å in the host lattice.^117^ Therefore, a review of the mechanism for the promising host Cs_2_NaYCl_6_ is recommended. Compared to the other compounds mentioned, Cs_2_NaYCl_6_ with an elpasolite-type structure is characterized by largely separated rare earth sites with a closest distance of about 7.59 Å.^118^ Since this compound fulfills many of the presented requirements for efficient ESA-based upconversion, we investigated its power-dependent blue-to-UV upconversion also at lower temperatures (77 K) to avoid temperature-induced decay processes of the intermediate ^3^P_0_ level based on its uncommonly low cut-off phonon energy of 287 cm^−1^ (Table 1 and Fig. S15†). Due to the lack of emission from the ^1^D_2_ level in Pr^3+^-activated Cs_2_NaYCl_6_ (Fig. 3), only the dependence on the emission of the ^3^P_0_ level and the 4f^1^5d^1^ states is considered.

**Table 4:** Characteristic dependencies of the population of the excited states of the Pr^3+^ ion (^1^D_2_, ^3^P_0_ and 4f^1^5d^1^) involved in the blue-to-UV upconversion process on the irradiated intensity *I*. A complete derivation can be found in the ESI

	ESA		ETU	
	Low pump rate	High pump rate	Low pump rate	High pump rate
^1^D_2_	∝*I*	∝*I*^−1^	∝*I*	∝Const.
^3^P_0_	∝*I*	∝Const.	∝*I*	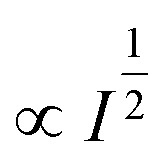
4f^1^5d^1^	∝*I*^2^	∝*I*	∝*I*^2^	∝*I*

**Fig. 9:**
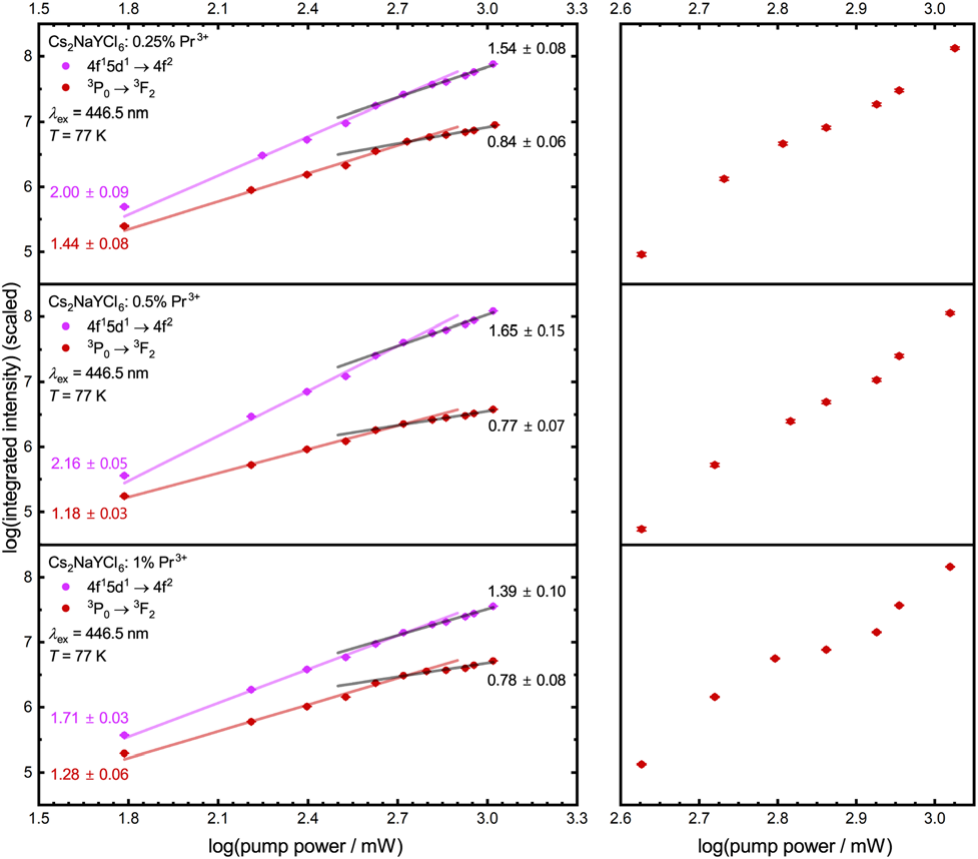
Double logarithmic plot of integrated upconversion and ^3^P_0_ intensity against laser pump power with linear regression for Cs_2_NaYCl_6_: 0.25 mol% Pr^3+^ (top), Cs_2_NaYCl_6_: 0.5 mol% Pr^3+^ (middle) and Cs_2_NaYCl_6_: 1 mol% Pr^3+^ (bottom) at 77 K. Intensity is scaled for comparison purposes. Slopes are given in the diagram. (Right) Integrated ^3^P_0_ intensity around the saturation point.

Under excitation with low incident power of the blue light source, the aforementioned slopes in the range of 2 for the UC luminescence and 1 for the luminescence of the intermediate ^3^P_0_ level are observed for Pr^3+^-activated Cs_2_NaYCl_6_ (Fig. 9, middle). After saturation, a drop in the slope is observed, with the power dependence of the intensity of the 4f^1^5d^1^-derived states decreasing to *n* = 1.65 ± 0.15 and the dependence of the intensity of ^3^P_0_ decreasing to *n* = 0.77 ± 0.07. As the slope of the 4f^1^5d^1^ upconverted emission intensity has not yet reached the anticipated value of *n* = 1 for an ESA mechanism, it can be assumed that saturation is not yet complete and therefore the slope of the ^3^P_0_ intensity will continue to decrease.

Since no precise statement can be made about the UC mechanism in Cs_2_NaYCl_6_: 0.5 mol% Pr^3+^ given the power limitations of our experimental setup, the measurements were repeated with a concentration of 0.25 mol% Pr^3+^ and 1 mol% Pr^3+^ (see the ESI† for XRPD and Rietveld refinement). A small change in the Pr^3+^ concentration in Cs_2_NaYCl_6_ does not lead to a decrease in the decay time of the ^3^P_0_ level indicating that cross-relaxation can be neglected at these low concentration levels in the chloridoelpasolite (see Fig. S24†). The slopes for the upconverted UV luminescence and the luminescence of the intermediate ^3^P_0_ level can be also reproduced in the other Pr^3+^-activated compounds in principle. However, a different behaviour is observed for the intensity of the ^3^P_0_ level after the commencement of saturation effects. After a drop in the slope at the saturation point, a renewed increase in intensity with power is observed shortly afterwards (see Fig. 9 right). According to Pollnau *et al.*,^116^ this behaviour is observable when ESA and ETU exist simultaneously, and was also reported for Re^4+^ in Cs_2_ZrCl_6_.^119,120^ This behaviour is observed in all Pr^3+^-activated chloridoelpasolites, but is very slight in the sample with 0.25 mol% and 0.5 mol% Pr^3+^. Consequently, it can be assumed that ETU plays a non-negligible role in Cs_2_NaYCl_6_: 1 mol% Pr^3+^. Similar conclusions have been anticipated for YSO:Pr^3+^.^78^

#### 2.2.4 Upconversion quantum yield *Φ*_UC_

For a direct comparison of the upconversion efficiency of ESA-based Pr^3+^-activated compounds, quantum yield measurements under blue light excitation were performed. It is important to note that upconversion quantum yields show a strong dependence on the excitation power.^54,59^ The experimental setup used in this work does not give absolute values for the upconversion quantum yield, but given equal measurement conditions for all regarded Pr^3+^-activated samples within this work, relative trends can be elucidated at least. Future studies need to be performed to find standardized, reproducible solutions to report reliable upconversion quantum yields in this challenging spectral range, as many standard integrating spheres and photodetectors have greatly reduced sensitivity in the deep-UV and may require UV-grade coatings or special detectors to achieve reasonable quantum efficiency. Furthermore, the upconverted UV signal is very weak in intensity and scattered laser light can produce a significant background in the UV detectors.

**Fig. 10:**
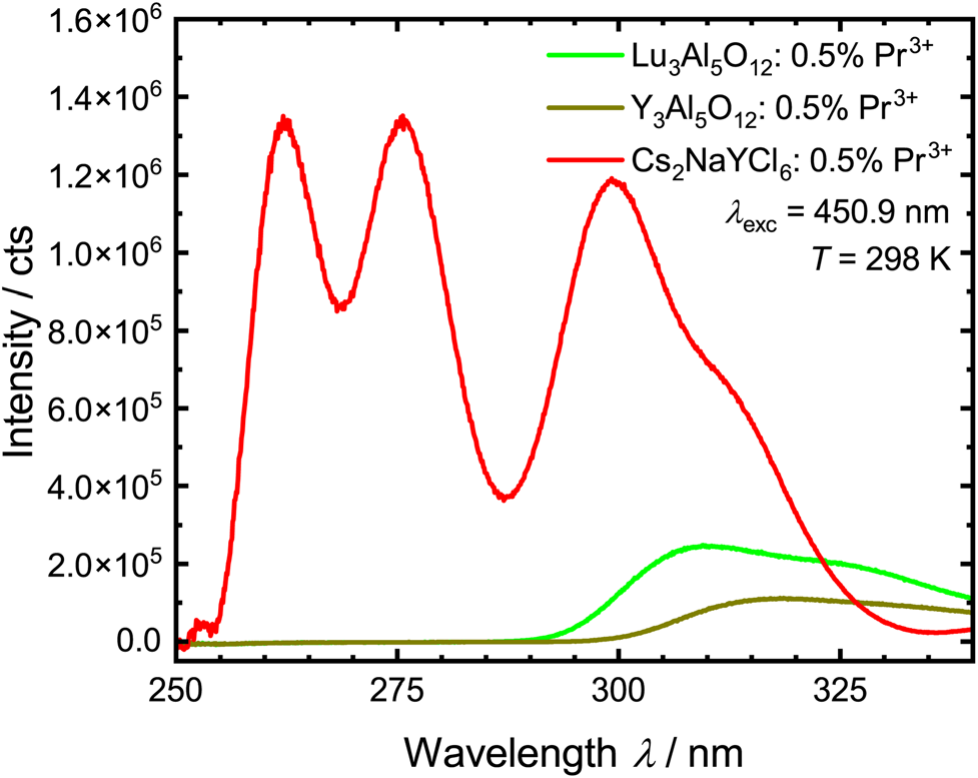
Comparison of the intensities of the UC luminescence of Pr^3+^ in LuAG, YAG and Cs_2_NaYCl_6_ measured under the same conditions (under air at 298 K) and with the same measurement parameters (1 nm emission bandwidth, 0.1 nm step size, 0.3 s dwell time).

The obtained upconversion quantum yields with the corresponding standard deviation for the Pr^3+^-activated compounds are given in Table 5. The VPL-450 used here has a power density of 0.59 W cm^−2^. This power density is therefore lower than the power density of modern LED chips, which can be up to 7.2 W cm^−2^.^121^ With the used integrating sphere setup, UC luminescence was only observed for Pr^3+^-activated YAG, LuAG and Cs_2_NaYCl_6_, with YAG:Pr^3+^ showing the lowest quantum yield of the three regarded compounds in the range of 0.009%. Based on these findings, it is anticipated that the quantum yields of the remaining compounds are lower than the one of YAG:Pr^3+^. The higher UC quantum yield of Cs_2_NaYCl_6_:Pr^3+^ compared to YAG:Pr^3+^ and LuAG:Pr^3+^ is also indicated in the intensity of the upconverted 4f^1^5d^1^ → 4f^2^-based emission spectra upon blue-light excitation recorded under the same conditions (Fig. 10). Furthermore, it is necessary to mention that Cs_2_NaYCl_6_:Pr^3+^ could not be measured in the ampoule and thus the hygroscopic properties could not be suppressed that would limit long-term applications. An even higher upconversion quantum yield is thus conceivable for Cs_2_NaYCl_6_:Pr^3+^.

**Table 5:** Results of quantum yield determination of the 0.5 mol% Pr^3+^-activated compounds considered within this work (*λ*_ex_ = 450.9 nm, *P* = 0.59 W cm^−2^). For missing UC luminescence, a quantum yield lower than Pr^3+^-activated YAG is given

	Upconversion quantum yield *Φ*_UC_/%
YAB:Pr^3+^	<0.009
NYB:Pr^3+^	<0.009
YPS:Pr^3+^	<0.009
YSO:Pr^3+^	<0.009
LuAG:Pr^3+^	0.025 ± 0.006
YAG:Pr^3+^	0.009 ± 0.001
V-YOF:Pr^3+^	<0.009
Cs_2_NaYCl_6_:Pr^3+^	0.105 ± 0.019

The UC quantum yields for the Pr^3+^-activated compounds are much lower than the ones known for the Yb^3+^/Er^3+^ UC couple, which can be up to *Φ*_UC_ = 2% (*P* = 0.6 W cm^−2^) in β-NaYF_4_.^54,56^

A better comparison of the UC quantum yields of Pr^3+^ and Yb^3+^/Er^3+^ than the value at the same power density is provided by the additional consideration of the photon flux *q*_p_, which depends on the power density *P* and the excitation wavelength *λ* of the used excitation source,5
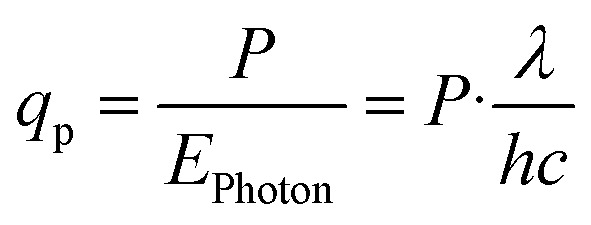


The corrected UC quantum yield thus results from the ratio of the photon flux, which equals the ratio of the excitation wavelengths at a given power density.6
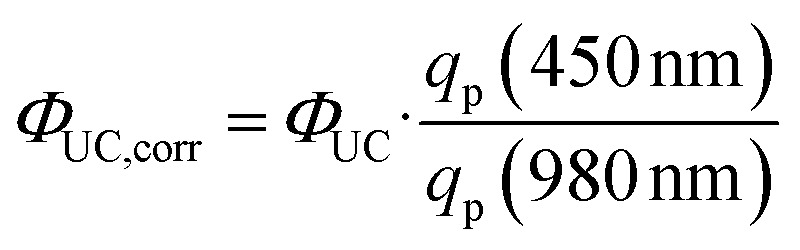


Taking this into account, the UC quantum yield of Yb^3+^/Er^3+^ in comparison to the one of Pr^3+^ is *Φ*_UC,corr_ = 0.92%, which is higher than the one of Pr^3+^-activated Cs_2_NaYCl_6_ with *Φ*_UC_ = 0.11%. This is consistent with the different UC mechanisms, as UC from Yb^3+^/Er^3+^ is known to dominantly occur *via* ETU, which shows a higher efficiency.^17^

**Fig. 11:**
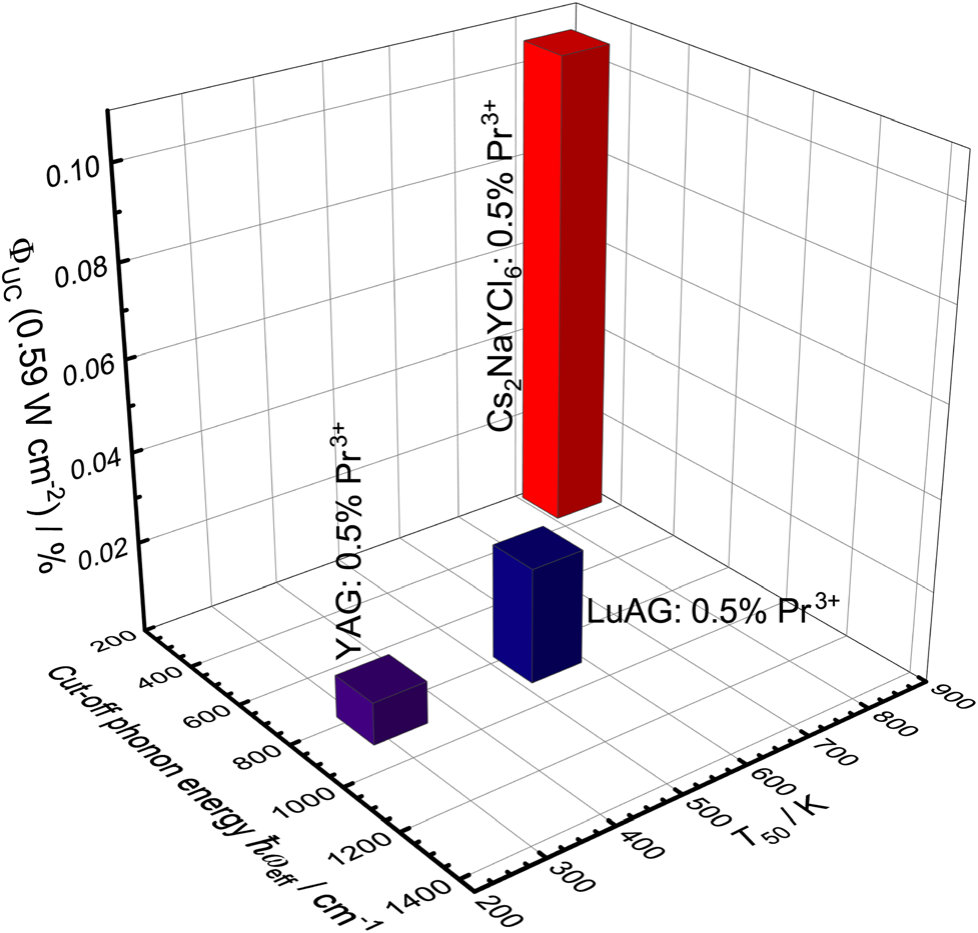
3D bar chart of the upconversion quantum yield in relation to the cut-off phonon energy of the host compound and the quenching temperature *T*_50_ of the 4f^1^5d^1^-based emission.

Although a UC quantum yield for Pr^3+^ could only be determined in three host compounds, the trend shows an increasing quantum yield with decreasing cut-off phonon energy and increasing quenching temperature *T*_50_ (Fig. 11). The influence of the quenching temperature *T*_50_ of the 4f^1^5d^1^-based emission becomes particularly evident when considering YAG and LuAG, as LuAG:Pr^3+^ has a UC quantum yield more than twice as large as YAG:Pr^3+^ despite similar phonon energies.

For the already well-studied Pr^3+^-activated YSO, no UC quantum yield could be determined with our setup. Previous work indicates a UC efficiency of 0.0019% (*P* = 1.65 mW cm^−2^)^122^ for YSO:Pr^3+^,Li^+^. As the UC efficiency for YSO:Pr^3+^,Li^+^ was determined *via* biodosimetry, no exact comparison can be made here. It is also noteworthy that the quantum yield of Pr^3+^-activated YSO seems to be lower despite a more efficient ETU mechanism for concentrations higher than 0.02 mol% of Pr^3+^.^78^ A UC quantum yield could also not be determined for Pr^3+^-activated YPS, which shows a higher UC efficiency than YSO:Pr^3+^ according to other studies.^75^ While the 4f^1^5d^1^-based emission in YSO shows slight quenching at room temperature, no decrease in the 4f^1^5d^1^ decay time is observed in YPS (Fig. 6). Consequently, the absence of UC luminescence in the quantum yield cannot be due to thermal quenching, which is also consistent with the power-dependent measurements (Fig. 7), but must be related to the high decay rate of the ^3^P_0_ level (Fig. 4 and Table 1).

A similarly low ESA-based UC efficiency of Pr^3+^ in the analysed silicates is also shown in YAB and NYB. For both Pr^3+^-activated compounds, no quantum yields can be measured with our setup, which is also due to the high decay rate of the ^3^P_0_ level (Fig. 4 and Table 1). Quenching of the 4f^1^5d^1^-based emission at room temperature can also be ruled out for both host compounds (Table 2 and Fig. 6). Thus, the Pr^3+^-activated borates are capable UV-emitting phosphors, but poor ESA-based blue-to-UV upconverters.

## 3 Conclusions

In this work, the different influences of host compounds on the excited-state absorption-based blue-to-UV upconversion of Pr^3+^ are presented. A high decay time of the intermediate state is required for this mechanism. Using time-resolved measurements, it is shown that the decay rate of the intermediate ^3^P_0_ level increases with increasing cut-off phonon energy of the host compound. This is consistent with the energy gap law and the resulting multiphonon relaxation into the energetically lower ^1^D_2_ level.

Furthermore, non-radiative decay of the excited 4f^1^5d^1^ states must be limited in order for them to exhibit a high quantum yield. This pathway is minimized if the thermal activation barrier to the crossover point with the low-energy 4f^2^ levels is high and thus only a small shift in the configurational coordinate is present. Rigid host compounds are suited choices to fulfill these requirements, as is the case in many borates, silicates and phosphates. Alternatively, this can also be achieved if Pr^3+^ substitutes smaller cation sites with yet high coordination numbers. Here, for example, a substitution of Lu^3+^, In^3+^ or Sc^3+^ would be suitable.

Those criteria fit especially well with the studied Pr^3+^-activated Cs_2_NaYCl_6_. With a small cut-off phonon energy *ℏω*_eff_ of 287 cm^−1^ and a quenching temperature *T*_50_ of 845 K, an upconversion quantum yield of *Φ*_UC_ = 0.11% for a power density of *P* = 0.59 W cm^−2^ is achieved. In direct comparison with the already known LuAG:Pr^3+^ and YPS:Pr^3+^, Cs_2_NaYCl_6_:Pr^3+^ thus shows the highest blue-to-UV efficiency for Pr^3+^-activated inorganic compounds reported to date.

The results of this work provide new guidelines for designing blue-to-UV phosphors with Pr^3+^ using ESA as a mechanism. The low quantum yield indicates that there are still limitations in the technical applications of Pr^3+^-activated solids for blue-to-UV ESA-based upconversion. Creating guidelines for designing Pr^3+^-activated solids that affect the ETU mechanism offers the possibility of further optimisation given the several orders of magnitude higher efficiency.

## Author contributions

T. Förster performed the measurements, planned the experiments, supervised data acquisition and curated the data. He performed quantum yield measurements and wrote the major part of the manuscript. J. Reifenberger, T. Moumin, and J. Helmbold synthesized relevant compounds, and acquired spectroscopic data. Ž. Antić, M. D. Dramićanin, and M. Suta conceptualized the project, acquired funding and reviewed the manuscript. T. Förster and M. Suta calculated the excited-state dynamics. M. Suta gave suggestions about the respective compounds, details in the synthesis and supervised the whole project. He reviewed and finalized the manuscript.

## Conflicts of interest

There are no conflicts to declare.

## Supplementary Material

SC-016-D5SC01862E-s001

## Data Availability

Source data generated in this study, which are presented in the main text and ESI,† are provided as Source Data files *via* the Zenodo repository under accession code https://doi.org/10.5281/zenodo.15589798. Source data are also available from the corresponding author upon request.
