# Supplementary material for: Design principles for (efficient) excited-state absorption-based blue-to-UV upconversion phosphors with Pr3+
Source: Chem Sci. 2025 Jun 2;16(27):12309–23. doi: 10.1039/d5sc01862e (PMC12147268; doi:10.1039/d5sc01862e)
Supplement: SC-016-D5SC01862E-s001 [file SC-016-D5SC01862E-s001.pdf]

Supplementary Information (SI) for Chemical Science.
This journal is © The Royal Society of Chemistry 2025

Design principles for (efficient) excited-state absorption-based blue-to-UV upconversion phosphors with Pr^{3+}

Tom Förster[a], Josefine Reifenberger[a], Tugce Moumin[a], Justus Helmbold[a], Željka Antić[b,c], Miroslav D. Dramićanin[b,c], Markus Suta[a]*

*** Corresponding author:**

Jun.-Prof. Dr. Markus Suta, Inorganic Photoactive Materials, Institute of Inorganic and Structural Chemistry, Heinrich Heine University Düsseldorf, Universitätsstraße 1, 40225 Düsseldorf, Germany; E-Mail: markus.suta@hhu.de

[a] T. Förster, J. Reifenberger, T. Moumin, J. Helmbold
Inorganic Photoactive Materials, Institute of Inorganic and Structural Chemistry, Heinrich Heine University, Universitätsstraße 1, 40225 Düsseldorf, Germany
E-Mail: tom.foerster@hhu.de

[b] Prof. Dr. Ž. Antić, Prof. Dr. M. D. Dramićanin
National Institute of Research and Development for Electrochemistry and Condensed Matter, INCEMC, Timisoara, Romania
E-Mail: zeljkaa@gmail.com, dramican@vinca.rs

[c] Prof. Dr. Ž. Antić, Prof. Dr. M. D. Dramićanin
Centre of Excellence for Photoconversion, Vinča Institute of Nuclear Sciences, National Institute of the Republic of Serbia, University of Belgrade, P.O. Box 522, 11351 Belgrade, Serbia
E-Mail: zeljkaa@gmail.com, dramican@vinca.rs

Contents

1 Experimental section **2**

1.1 Chemical reagents 2

1.2 Synthesis of activated $YAl_3(BO_3)_4$ 2

1.3 Synthesis of activated $Na_3Y(BO_3)_2$ 2

1.4 Synthesis of activated β-$Y_2Si_2O_7$ 3

1.5 Synthesis of activated X2-Y_2SiO_5 3

1.6 Synthesis of activated $Y_3Al_5O_{12}$ and $Lu_3Al_5O_{12}$ 3

1.7 Synthesis of activated $Y_7O_6F_9$ 4

1.8 Synthesis of activated Cs_2NaYCl_6 4

1.9 Characterization and methods 4

2 Rietveld refinements **6**

3 Luminescence spectra and decay times **13**

4 IR spectra **17**

5 Upconversion luminescence **21**

6 Decay curves of $Cs_2NaY_{1\text{-}x}Pr_xCl_6$, (x = 0.005, 0.01) **25**

7 Rate equations of the upconversion mechanism **26**

1 Experimental section

1.1 Chemical reagents

All raw materials of Y_2O_3 (ChemPur, 99.999 % REO), Lu_2O_3 (ChemPur, 99.999 % REO), Pr_6O_{11} (Thermo Fischer Scientific, 99.996 % REO), HNO_3 (Sigma Aldrich, ≥65 %), H_2O_2 (Supelco, 30 %), $Al(NO_3)_3 \cdot 9\ H_2O$ (ChemPur, 99.998 %), NH_3 (VWR, 25 %), tetraethyl orthosilicate (Thermo Fischer Scientific, ≥99 %), urea (Grüssing, ≥99.5 %), H_3BO_3 (ChemSolute, ≥99.5 %), $(NH_4)_2CO_3$ (Thermo Fischer Scientific, 31.5 %), Na_2CO_3 (Grüssing, ≥99.5 %), NH_4F (Thermo Fischer Scientific, ≥98 %), CsCl (Thermo Fischer Scientific, 99.99 % metal basis), NaCl (Sigma Aldrich, ≥99.5 %), HCl (Sigma Aldrich, ≥37 %) were used without additional purification. Due to the high cross relaxation processes and for suppression of energy transfer upconversion in highly activated Pr^{3+} compounds[1–3], nominal activator concentrations were set to 0.5 mol % with respect to the Y or Lu content, respectively. For synthesizing the activated phosphors, a stochiometric amount of Y_2O_3/Lu_2O_3 was omitted.

1.2 Synthesis of activated $YAl_3(BO_3)_4$

Microcrystalline powder of Pr^{3+}-activated YAB ($Y_{0.995}Pr_{0.005}Al_3(BO_3)_4$) was prepared by a modified urea-nitrate solution-based combustion route[4] as described elsewhere.[5] Rare earth nitrates were generated by dissolving the oxides in concentrated HNO_3 (v% = 67 %). The solution was evaporated and refilled with dist. H_2O until a pH of 7 was reached. Then $Al(NO_3)_3 \cdot 9\ H_2O$, H_3BO_3 and urea were added to the solution. The clear solution was stirred for 30 min. After that, the solution was put in a preheated 500 °C muffle furnace for 10 min. The resulting colorless powder was ground with H_3BO_3 and sintered for 17 h at 1100 °C in a muffle furnace.

1.3 Synthesis of activated $Na_3Y(BO_3)_2$

Microcrystalline powder of Pr^{3+}-activated NYB ($Na_3Y_{0.995}Pr_{0.005}(BO_3)_2$) was prepared by a modified co-precipitation method as described elsewhere[6]. Y_2O_3 and Pr_6O_{11} were dissolved in concentrated HNO_3 (v% = 67 %). A few drops of a concentrated H_2O_2 (v% = 67 %) solution were added in order to reduce Pr^{4+} to Pr^{3+}. A saturated solution of $(NH_4)_2CO_3$ was added resulting in a colorless precipitate, which was filtered, washed with dist. H_2O and ethanol and dried at 80 °C for 16 h. The dried precipitate was ground with a stochiometric amount of Na_2CO_3 and a 3 w% excess of H_3BO_3. The resulting powder was sintered for 18 h at 900 °C.

1.4 Synthesis of activated β-$Y_2Si_2O_7$

Microcrystalline powder of Pr^{3+}-activated YPS (β-$Y_{1.99}Pr_{0.01}Si_2O_7$) was prepared by a sol-gel combustion synthesis. Stochiometric amounts of Y_2O_3 and Pr_6O_{11} were dissolved in concentrated HNO_3 (v% = 67 %). A few drops of a concentrated H_2O_2 (v% = 30 %) solution were added in order to reduce all Pr^{4+} to Pr^{3+}. The solvent was repeatedly evaporated and the solid residue re-dissolved in dist. H_2O until a pH of 7 was reached. Then, ethanol and a stochiometric excess of tetraethyl orthosilicate were added. After that urea was added to the solution. The solution was evaporated until a transparent gel was formed. The gel was dried at 80 °C overnight and then calcined at 510 °C for 1.5 h. The resulting colorless powder was then ground and calcined at 1350 °C for 24 h. The powder was ground again and then calcined for additional 14 h at 1350 °C.

1.5 Synthesis of activated X2-Y_2SiO_5

Microcrystalline powder of Pr^{3+}-activated YSO (X2-$Y_{1.99}Pr_{0.01}SiO_5$) was prepared by a sol-gel synthesis. Stochiometric amounts of Y_2O_3 and Pr_6O_{11} were dissolved in nitric acid. A few drops of a concentraed H_2O_2 (v% = 67 %) solution were added in order to reduce all Pr^{4+} to Pr^{3+}. The solution was repeatedly evaporated and the remaining residue re-dissolved in dist. H_2O until a pH of 7 was reached. Then, ethanol and a stochiometric amount of tetraethyl orthosilicate were added. The solution was stirred for 30 minutes and evaporated, forming a transparent gel. The gel was dried at 80 °C overnight and ground to a fine powder before final calcination 1350 °C for 12 h.

1.6 Synthesis of activated $Y_3Al_5O_{12}$ and $Lu_3Al_5O_{12}$

Microcrystalline powder of Pr^{3+}-activated YAG ($Y_{2.985}Pr_{0.015}Al_5O_{12}$) and Pr^{3+}-activated LuAG ($Lu_{2.985}Pr_{0.015}Al_5O_{12}$) were synthesized by a modified co-precipitation method as described elsewhere[7]. Stochiometric amounts of Y_2O_3/Lu_2O_3 and Pr_6O_{11} were dissolved in concentrated HNO_3 (v% = 67 %). A few drops of a concentrated H_2O_2 (v% = 30 %) solution were added in order to reduce Pr^{4+} to Pr^{3+}. The solution was repeatedly evaporated and the remaining residue re-dissolved in dist. H_2O until a pH of 7 was reached. Then a stochiometric amount of $Al(NO_3)_3 \cdot 9\ H_2O$ was added to the solution. NH_3 was added dropwise, resulting in a colorless, gelatinous precipitate. It was washed repeatedly with dist. H_2O and ethanol and dried overnight at 80 °C. The obtained colorless powder was ground and pre-calcined at 400 °C for 1 h before calcining at 1300 °C for additional 3 h.

1.7 Synthesis of activated $Y_7O_6F_9$

Microcrystalline powder of Pr^{3+}-activated V-YOF ($Y_{6.965}Pr_{0.035}O_6F_9$) was prepared by a solid-state reaction. Stochiometric amounts of Y_2O_3 and Pr_6O_{11} were ground with an excess of NH_4F (ratio 1:2 RE^{3+}:F^-) and calcined at 1050 °C for 2 h with intermediate grinding after every 0.5 h.

1.8 Synthesis of activated Cs_2NaYCl_6

Microcrystalline powder of Pr^{3+}-activated Cs_2NaYCl_6 ($Cs_2NaY_{1\text{-}x}Pr_xCl_6$, x = 0.0025, 0.005, 0.01) was prepared by evaporating a solution containing the appropiate cations in hydrochloric acid as described elsewhere[8]. For that purpose, Y_2O_3, Pr_6O_{11}, CsCl and NaCl were dissolved in concentrated HCl (v% = 37 %) with a few drops of a concentrated H_2O_2 (v% = 30 %) solution and the solution was evaporated to dryness. The resulting white powder was then washed with ethanol several times. Due to the hygroscopicity of the obtained powder[8,9], it was enclosed in a evacuated thin, optically transparent quartz ampoule (ilmasil® quality, *QSIL GmbH*) right after synthesis and powder diffraction measurement.

1.9 Characterization and methods

Sample purity was verified by powder X-ray diffraction using a *Malvern Panalytical* X'pert Pro powder diffractometer with Cu K_α radiation in a Bragg-Brentano geometry and reflection. XRPD data was modeled using Rietveld refinement with TOPAS 7[10].

MIR spectra were recorded with a *PerkinElmer* Spectrum Two FT-IR spectrometer equipped with a $LiTaO_3$ detector and an ATR unit. For IR spectra below 400 cm^{-1} a *PerkinElmer* Frontier FIR-IR spectrometer was used.

A FLS1000 photoluminescence spectrometer from *Edinburgh Instruments* was used for optical measurements. It was equipped with a 450 W Xe arc lamp for excitation, double excitation and emission monochromators in Czerny-Turner configuration and a thermoelectrically cooled (−20 °C) photomultiplier tube PMT-980 (Hamamatsu). Emission spectra were corrected with respect to the grating efficiency and PMT sensitivity, while excitation spectra were additionally corrected with respect to the lamp intensity. Upconversion spectra were only corrected by PMT sensitivity due to overcorrection. For power-dependent photoluminescence spectra a MDL-F-450-1W laser diode (PhotonTec, λ = 446.5 nm) with an adjustable power (10 mW ... 1000 mW) was used as excitation source. The output power was adjusted after the measurement using a correction function, which was generated by cross-checking the nominal power with a power meter (Coherent FieldmaxII equipped with a Coherent PS19 power sensor).

Photoluminescence decay traces of the $4f^15d^1 \rightarrow 4f^2$-based emission were recorded on a FS5 photoluminescence spectrometer from *Edinburgh Instruments* using an EPLED-

250 (*Edinburg Instruments*, 40 µW average peak power, $\lambda = 257.1$ nm, $\Delta\tau = 718$ ps temporal pulse width) and an EPLED-270 (*Edinburg Instruments*, 40 µW average peak power, $\lambda = 278.8$ nm, $\Delta\tau = 747.6$ ps temporal pulse width) for excitation using time-correlated single-photon counting (TCSPC) as the detection mode. The signal count rate was kept below 10 % of the repetition rate to avoid pile-up effects. For time-resolved measurements on the $4f^2 \rightarrow 4f^2$-based emission, a VPL-450 (*Edinburgh Instruments*, 53 mW average peak power in CW mode, $\lambda = 450.9$ nm) with adjustable temporal pulse width (0.1 µs ... 1 ms) and a variable repetition rate (0.1 Hz ... 5 MHz) was used as excitation source using single-photon mulitchannel scaling (MCS) as the detection mode. Average decay times were estimated using an intensity-weighting scheme:

$$\langle\tau\rangle = \frac{A_1\tau_1^2 + ... + A_n\tau_n^2}{A_1\tau_1 + ... + A_n\tau_n} \tag{1}$$

Temperature-dependent measurements were performed with a Linkam Scientific THMS600 temperature cell with an accuracy of ±0.1 °C in the temperature range between −196 °C (liquid nitrogen) and 500 °C. Constant temperature intervals of $\Delta T = 25$ °C were set for temperature-dependent measurements.

Absolute quantum yields of the upconversion luminescence at room temperature were measured with a BenFlect®-coated integrating sphere using the VPL-450 in CW mode for excitation. The irradiated area was estimated to be 9 mm^2 giving a power density about 0.59 W cm^{-2}.

The scattering light $I(\lambda)_{\text{observed}}$ was measured using a NDUV240B neutral density filter (*O.D.* = 4, *Thorlabs*) to increase the accuracy in the comparably low upconversion emission intensity assessment and corrected for the transmission of the neutral density filter $T(\lambda)_{\text{filter}}$[11]:

$$I(\lambda)_{\text{corrected}} = \frac{I(\lambda)_{\text{observed}}}{T(\lambda)_{\text{filter}}} \tag{2}$$

To check the measured values, measurements were carried out according to the method described by Schröder *et al.*[11] A FGUV5S bandpass filter (*Thorlabs*) was used in front of the detector. To measure over the excitation range, an additional NDUV230B neutral density filter (*O.D.* = 3, *Thorlabs*) was placed between the excitation source and the sample. The measured intensities were corrected by the corresponding transmissions of the respective filters (the excitation range was therefore corrected by both filters) for evaluation. The values obtained in this way matched within the margin of error.

2 Rietveld refinements

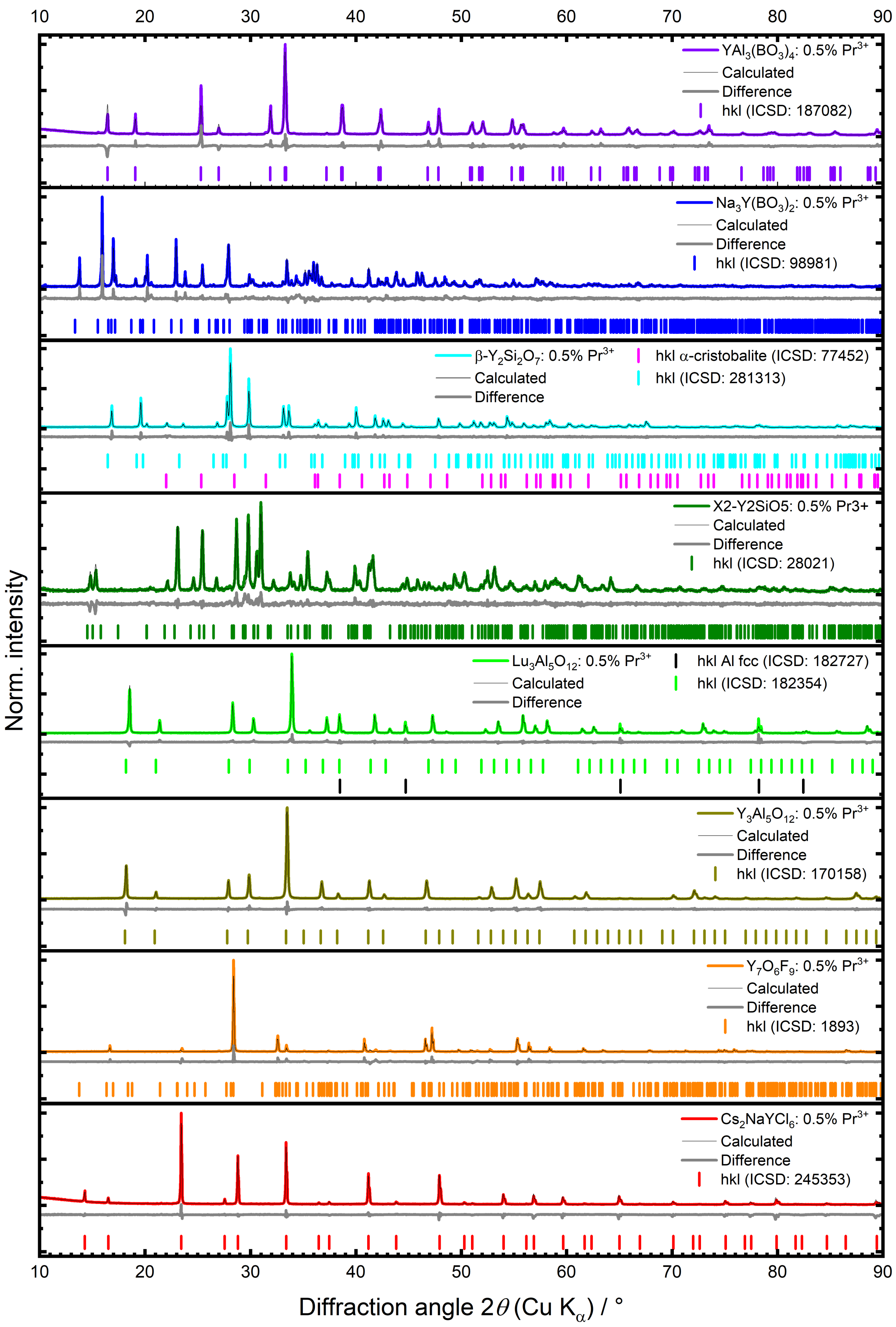

Figure S1: Rietveld-refined XRPD patterns of the synthesized materials activated with 0.5 mol % Pr^{3+}.

Table S1: Cell parameters and refinement parameters of YAB:Pr^{3+} obtained from Rietveld refinement compared to single-crystal structural data of YAB (ICSD repository no. 20223)[12].

	**YAB:Pr^{3+}**	**YAB (ICSD: 20223)**
**Crystal system**	trigonal	trigonal
**Space group**	$R32$ (155)	$R32$ (155)
$\boldsymbol{a}$ **/ Å**	9.2903(0)	9.295
$\boldsymbol{b}$ **/ Å**	9.2903(0)	9.295
$\boldsymbol{c}$ **/ Å**	7.2405(0)	7.243
α / °	90	90
β / °	90	90
γ / °	120	120
**Cell volume / Å^3**	541.198	541.94
$\boldsymbol{R_{Bragg}}$ **/ %**	8.79	-
$\boldsymbol{R_{exp}}$ **/ %**	3.00	-
$\boldsymbol{R_{wp}}$ **/ %**	11.60	-
$\boldsymbol{R_{p}}$ **/ %**	7.52	-
G.o.o.F.	3.86	-

Table S2: Cell parameters and refinement parameters of NYB:Pr^{3+} obtained from Rietveld refinement compared to polycrystalline powder structural data of NYB (ICSD depository no.: 94981)[13].

	**NYB:Pr^{3+}**	**NYB (ICSD: 94981)**
**Crystal system**	monoclinic	monoclinic
**Space group**	$P2_1/c$ (14)	$P2_1/c$ (14)
$\boldsymbol{a}$ **/ Å**	6.5053(0)	6.505
$\boldsymbol{b}$ **/ Å**	8.5176(0)	8.5172
$\boldsymbol{c}$ **/ Å**	12.0215(4)	12.0213
α / °	90	90
β / °	118.73	118.73
γ / °	90	90
**Cell volume / Å^3**	585.772	584.04
$\boldsymbol{R_{Bragg}}$ **/ %**	8.02	-
$\boldsymbol{R_{exp}}$ **/ %**	6.50	-
$\boldsymbol{R_{wp}}$ **/ %**	13.53	-
$\boldsymbol{R_{p}}$ **/ %**	10.07	-
G.o.o.F.	2.08	-

Table S3: Cell parameters and refinement parameters of YPS:Pr^{3+} obtained from Rietveld refinement compared to single-crystal structural data of YPS (ICSD depository no.: 281313)[14].

	**YPS:Pr^{3+}**	**YPS (ICSD: 281313)**
**Crystal system**	monoclinic	monoclinic
**Space group**	$C2/m$ (12)	$C2/m$ (12)
$\boldsymbol{a}$ **/ Å**	6.8694(3)	6.8667
$\boldsymbol{b}$ **/ Å**	8.9630(4)	8.959
$\boldsymbol{c}$ **/ Å**	4.7127(0)	4.7167
α / °	90	90
β / °	101.724	101.724
γ / °	90	90
**Cell volume / Å^3**	284.077	284.11
$\boldsymbol{R_{\mathrm{Bragg}}}$ **/ %**	2.83	-
$\boldsymbol{R_{\mathrm{exp}}}$ **/ %**	17.22	-
$\boldsymbol{R_{\mathrm{wp}}}$ **/ %**	10.88	-
$\boldsymbol{R_{\mathrm{p}}}$ **/ %**	8.60	-
G.o.o.F.	0.63	-

Table S4: Cell parameters and refinement parameters of YSO:Pr^{3+} obtained from Rietveld refinement compared to crystal structural data of YSO (ICSD depository no.: 28021)[15].

	**YSO:Pr^{3+}**	**YSO (ICSD: 28021)**
**Crystal system**	monoclinic	monoclinic
**Space group**	$C2/c$ (15)	$C2/c$ (15)
$\boldsymbol{a}$ **/ Å**	10.4210(5)	10.41
$\boldsymbol{b}$ **/ Å**	6.7261(8)	6.721
$\boldsymbol{c}$ **/ Å**	12.4924(0)	12.49
α / °	90	90
β / °	102.65	102.65
γ / °	90	90
**Cell volume / Å^3**	854.177	852.66
$\boldsymbol{R_{\mathrm{Bragg}}}$ **/ %**	4.62	-
$\boldsymbol{R_{\mathrm{exp}}}$ **/ %**	8.28	-
$\boldsymbol{R_{\mathrm{wp}}}$ **/ %**	12.36	-
$\boldsymbol{R_{\mathrm{p}}}$ **/ %**	9.42	-
G.o.o.F.	1.49	-

Table S5: Cell parameters and refinement parameters of LuAG:Pr^{3+} obtained from Rietveld refinement compared to polycrystalline powder structural data of LuAG (ICSD depository no.: 182354)[16].

	**LuAG:Pr^{3+}**	**LuAG (ICSD: 182354)**
**Crystal system**	cubic	cubic
**Space group**	$Ia\bar{3}d$ (230)	$Ia\bar{3}d$ (230)
$\boldsymbol{a}$ / Å	11.9362(3)	11.9361
$\boldsymbol{b}$ / Å	11.9362(3)	11.9361
$\boldsymbol{c}$ / Å	11.9362(3)	11.9361
α / °	90	90
β / °	90	90
γ / °	90	90
**Cell volume / $Å^3$**	1700.598	1700.54
$\boldsymbol{R_{\mathrm{Bragg}}}$ / %	4.88	-
$\boldsymbol{R_{\mathrm{exp}}}$ / %	4.28	-
$\boldsymbol{R_{\mathrm{wp}}}$ / %	10.31	-
$\boldsymbol{R_{\mathrm{p}}}$ / %	7.51	-
G.o.o.F.	2.41	-

Table S6: Cell parameters and refinement parameters of YAG:Pr^{3+} obtained from Rietveld refinement compared to single-crystal structural data of YAG (ICSD depository no.: 170158)[17].

	**YAG:Pr^{3+}**	**YAG (ICSD: 170158)**
**Crystal system**	cubic	cubic
**Space group**	$Ia\bar{3}d$ (230)	$Ia\bar{3}d$ (230)
$\boldsymbol{a}$ / Å	12.0026(4)	12.0003
$\boldsymbol{b}$ / Å	12.0026(4)	12.0003
$\boldsymbol{c}$ / Å	12.0026(4)	12.0003
α / °	90	90
β / °	90	90
γ / °	90	90
**Cell volume / $Å^3$**	1729.142	1728.13
$\boldsymbol{R_{\mathrm{Bragg}}}$ / %	1.75	-
$\boldsymbol{R_{\mathrm{exp}}}$ / %	3.63	-
$\boldsymbol{R_{\mathrm{wp}}}$ / %	7.72	-
$\boldsymbol{R_{\mathrm{p}}}$ / %	5.93	-
G.o.o.F.	2.13	-

Table S7: Cell parameters and refinement parameters of V-YOF:Pr^{3+} obtained from Rietveld refinement compared to single-crystal structural data of V-YOF (ICSD depository no.: 1893)[18].

	**V-YOF:Pr^{3+}**	**V-YOF (ICSD: 1893)**
**Crystal system**	orthorhombic	orthorhombic
**Space group**	*Abm*2 (39)	*Abm*2 (39)
$\boldsymbol{a}$ **/ Å**	5.3996(0)	5.42
$\boldsymbol{b}$ **/ Å**	38.7705(1)	38.58
$\boldsymbol{c}$ **/ Å**	5.5301(0))	5.527
α / °	90	90
β / °	90	90
γ / °	90	90
**Cell volume / Å^3**	1157.692	1155.72
$\boldsymbol{R_{Bragg}}$ **/ %**	10.13	-
$\boldsymbol{R_{exp}}$ **/ %**	4.65	-
$\boldsymbol{R_{wp}}$ **/ %**	16.89	-
$\boldsymbol{R_{p}}$ **/ %**	10.91	-
G.o.o.F.	3.63	-

Table S8: Cell parameters and refinement parameters of Cs_2NaYCl_6: 0.5 mol % Pr^{3+} obtained from Rietveld refinement compared to single-crystal structural data of Cs_2NaYCl_6 (ICSD depository no.: 245353)[19].

	**Cs_2NaYCl_6: 0.5 mol % Pr^{3+}**	**Cs_2NaYCl_6 (ICSD: 245353)**
**Crystal system**	cubic	cubic
**Space group**	$Fm\bar{3}m$ (225)	$Fm\bar{3}m$ (225)
$\boldsymbol{a}$ **/ Å**	10.7279(0)	10.7275
$\boldsymbol{b}$ **/ Å**	10.7279(0)	10.7275
$\boldsymbol{c}$ **/ Å**	10.7279(0)	10.7275
α / °	90	90
β / °	90	90
γ / °	90	90
**Cell volume / Å^3**	1234.643	1234.51
$\boldsymbol{R_{Bragg}}$ **/ %**	13.08	-
$\boldsymbol{R_{exp}}$ **/ %**	4.57	-
$\boldsymbol{R_{wp}}$ **/ %**	12.63	-
$\boldsymbol{R_{p}}$ **/ %**	8.24	-
G.o.o.F.	2.76	-

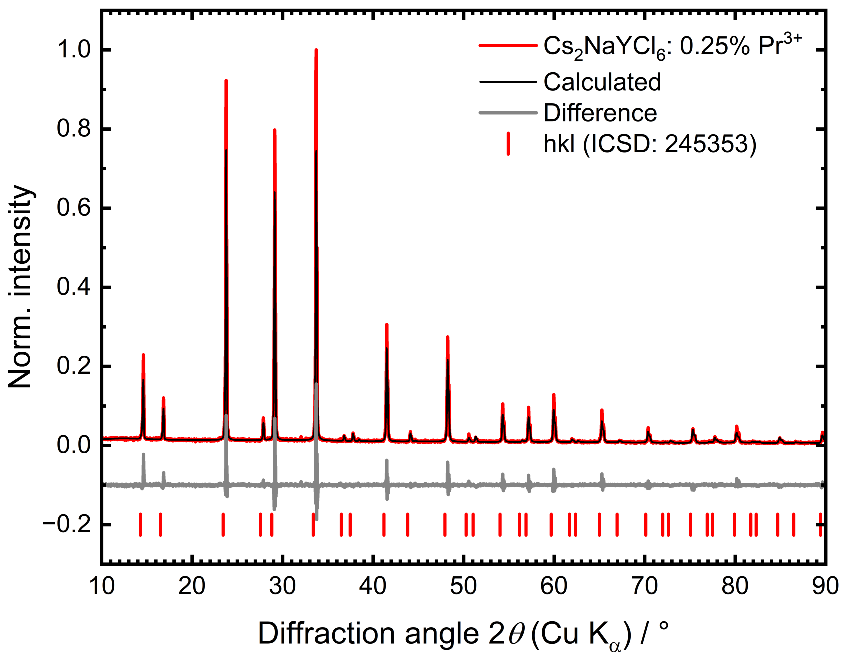

Figure S2: Rietveld-refined XRPD pattern of Cs_2NaYCl_6 activated with 0.25 mol % Pr^{3+}.

Table S9: Cell parameters and refinement parameters of Cs_2NaYCl_6: 0.25 mol % Pr^{3+} obtained from Rietveld refinement compared to single-crystal structural data of Cs_2NaYCl_6 (ICSD depository no.: 245353)[19].

	**Cs_2NaYCl_6: 0.25 mol % Pr^{3+}**	**Cs_2NaYCl_6 (ICSD: 245353)**
**Crystal system**	cubic	cubic
**Space group**	$Fm\bar{3}m$ (225)	$Fm\bar{3}m$ (225)
$\boldsymbol{a}$ / Å	10.7278(1)	10.7275
$\boldsymbol{b}$ / Å	10.7278(1)	10.7275
$\boldsymbol{c}$ / Å	10.7278(1)	10.7275
α / °	90	90
β / °	90	90
γ / °	90	90
**Cell volume / $Å^3$**	1234.631	1234.51
R_{Bragg} / %	4.35	-
R_{exp} / %	6.94	-
R_{wp} / %	13.75	-
R_p / %	10.65	-
G.o.o.F.	1.98	-

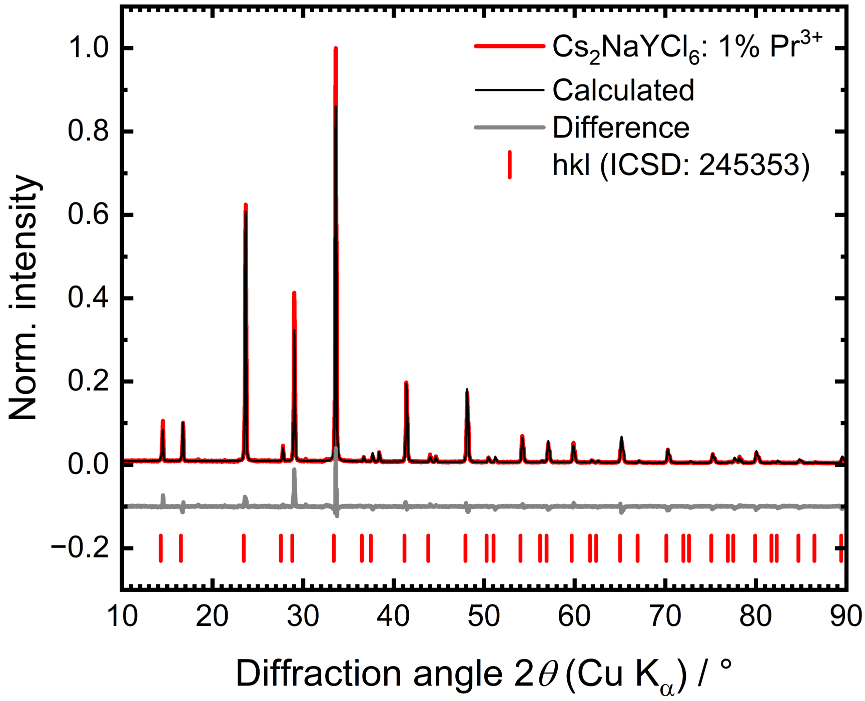

Figure S3: Rietveld-refined XRPD pattern of Cs_2NaYCl_6 activated with 1 mol % Pr^{3+}.

Table S10: Cell parameters and refinement parameters of Cs_2NaYCl_6: 1 mol % Pr^{3+} obtained from Rietveld refinement compared to single-crystal structural data of Cs_2NaYCl_6 (ICSD depository no.: 245353)[19].

	**Cs_2NaYCl_6: 1 mol % Pr^{3+}**	**Cs_2NaYCl_6 (ICSD: 245353)**
**Crystal system**	cubic	cubic
**Space group**	$Fm\bar{3}m$ (225)	$Fm\bar{3}m$ (225)
$\boldsymbol{a}$ **/ Å**	10.7287(1)	10.7275
$\boldsymbol{b}$ **/ Å**	10.7287(1)	10.7275
$\boldsymbol{c}$ **/ Å**	10.7287(1)	10.7275
α / °	90	90
β / °	90	90
γ / °	90	90
**Cell volume / Å^3**	1234.930	1234.51
$\boldsymbol{R_{Bragg}}$ **/ %**	7.36	-
$\boldsymbol{R_{exp}}$ **/ %**	5.49	-
$\boldsymbol{R_{wp}}$ **/ %**	12.08	-
$\boldsymbol{R_p}$ **/ %**	8.94	-
G.o.o.F.	2.20	-

3 Luminescence spectra and decay times

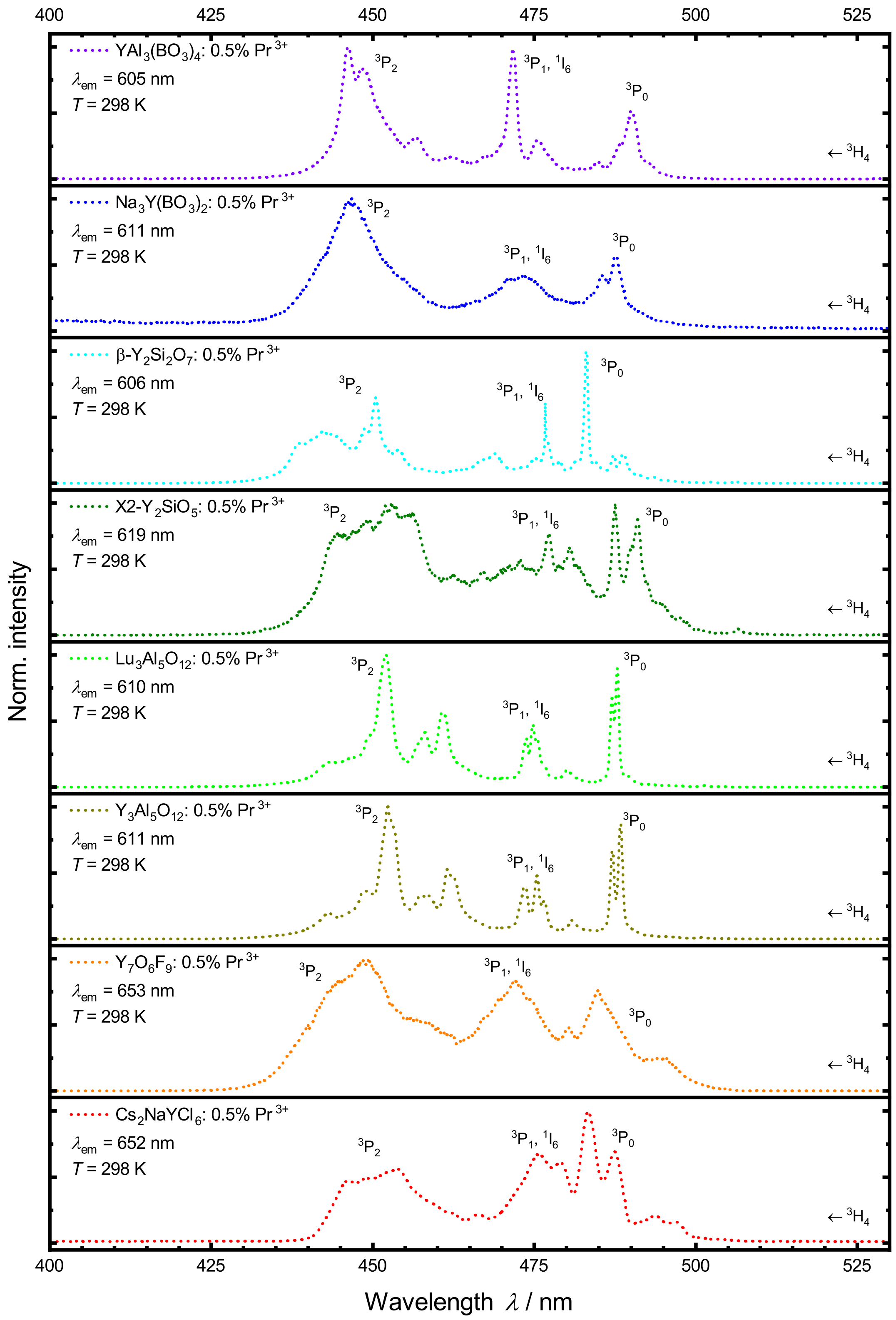

Figure S4: Normalized photoluminescence excitation spectra of the synthesized powders at 298 K, monitoring the emission maximum around 650 nm. The spectra show the ^3P_J (J = 0, 1, 2), $^1I_6 \leftarrow {}^3H_4$ excitation.

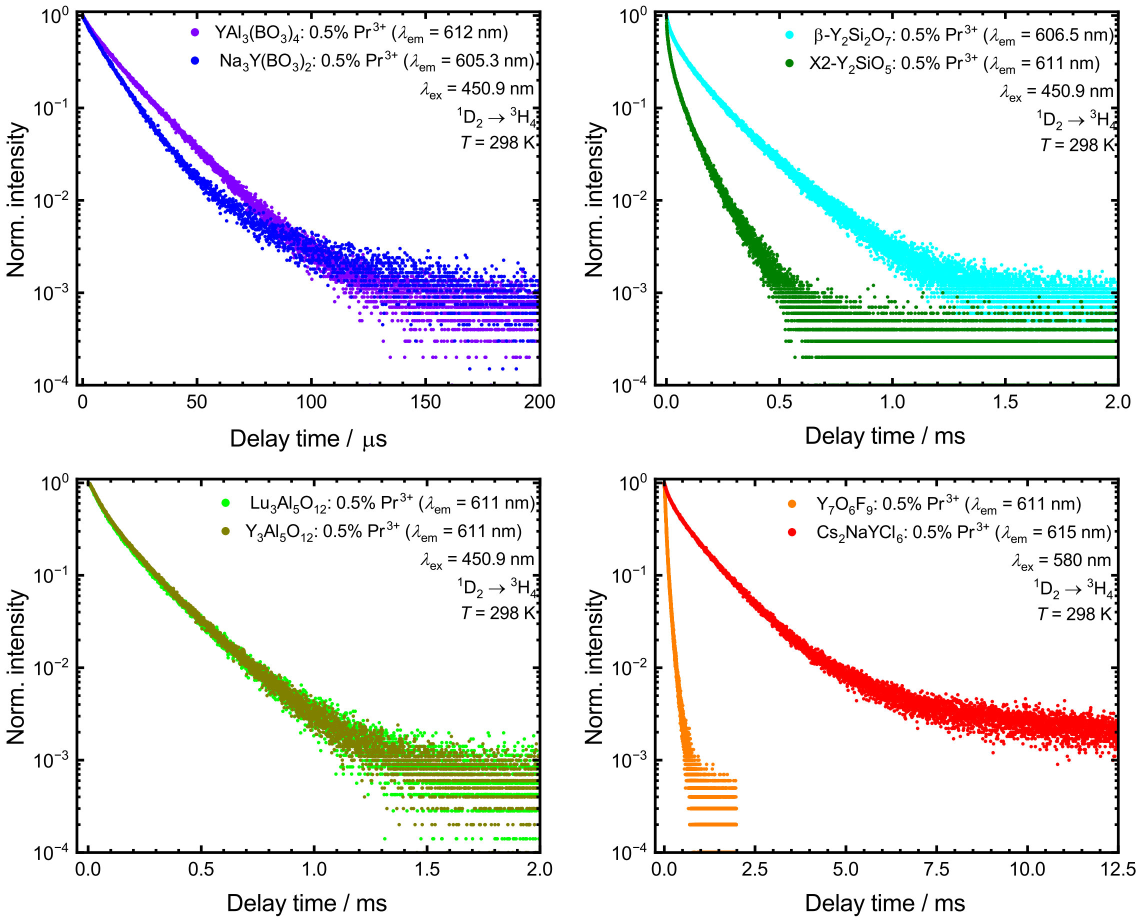

Figure S5: Photoluminescence decay curves of the synthesized samples of the $^1D_2$ level at 298 K monitoring the $^1D_2 \rightarrow {}^3H_4$-based emission around 600 nm. Due to strongly differing decay times, decay curves are shown for different delay times. Values of the measured decay times are given in Table 1 in the main manuscript.

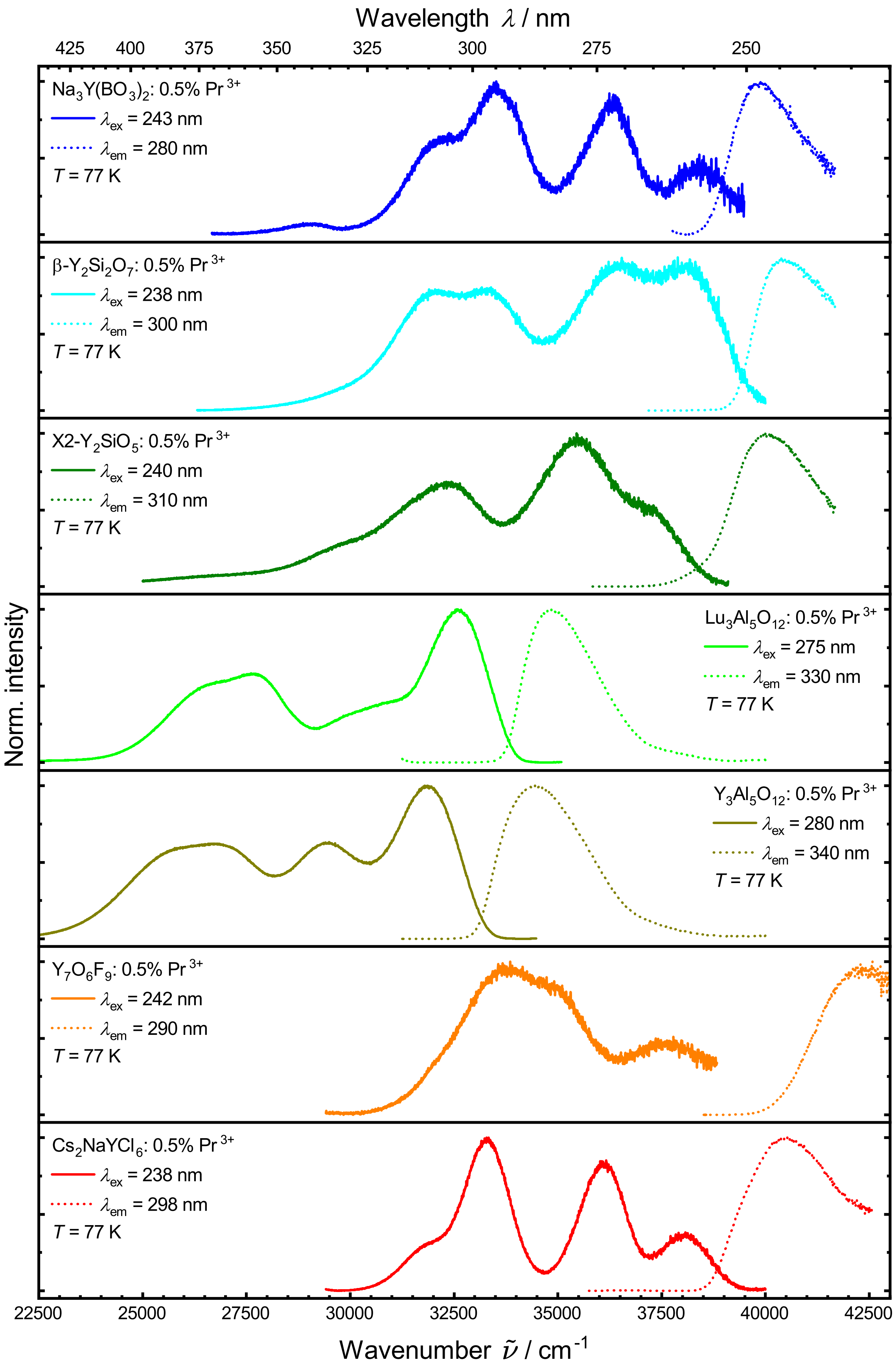

Figure S6: Normalized photoluminescence emission (solid lines) and excitation spectra (dotted lines) of the synthesized samples at 77 K in UV range showing emission and excitation of $4f^1 5d^1$ states. Spectra were measured against wavelengths and converted to wavenumbers using a Jacobian transformation[20].

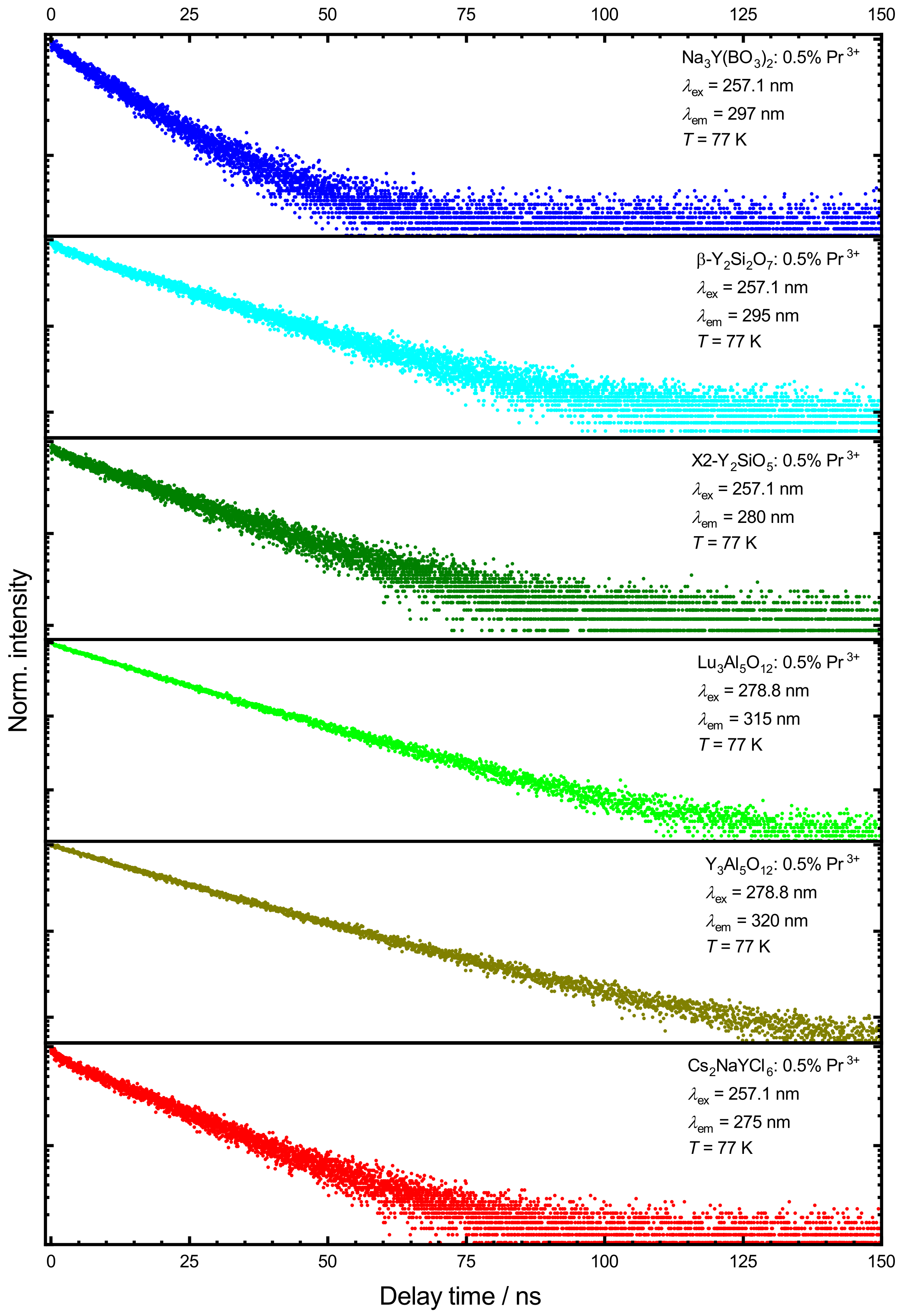

Figure S7: Decay curves of the $4f^15d^1$ states of the synthesized samples at 77 K in the range of nanoseconds. For the garnets, an EPLED-270 was used as excitation source. For the other samples, an EPLED-250 was used. Decay curves of Pr^{3+} in YAB and V-YOF are not depicted because of limitation in the experimental setup.

4 IR spectra

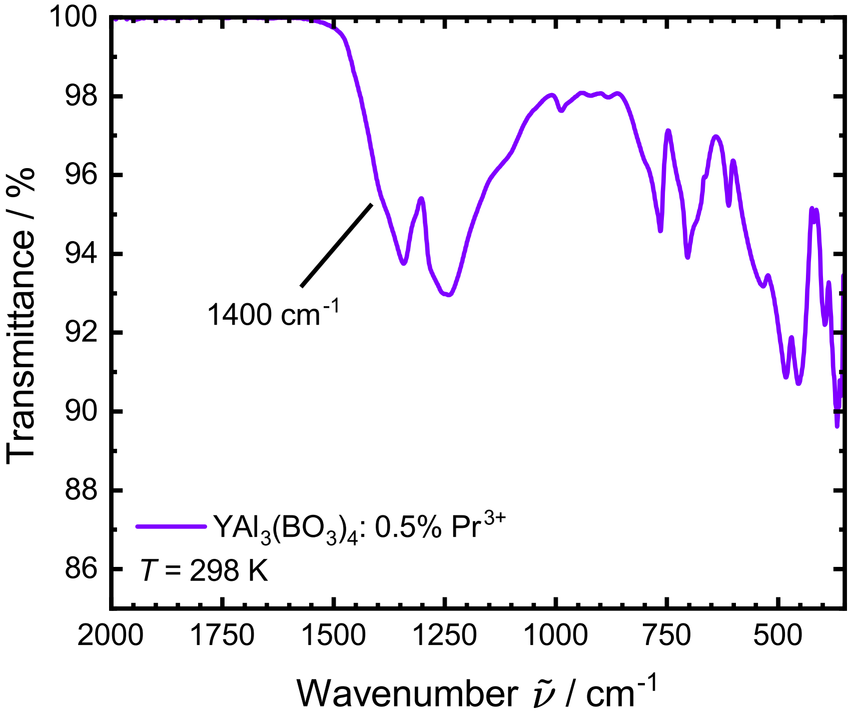

Figure S8: Infrared spectrum of the Pr^{3+}-activated $YAl_3(BO_3)_4$ at room temperature. The cut-off phonon energy is given in the spectrum.

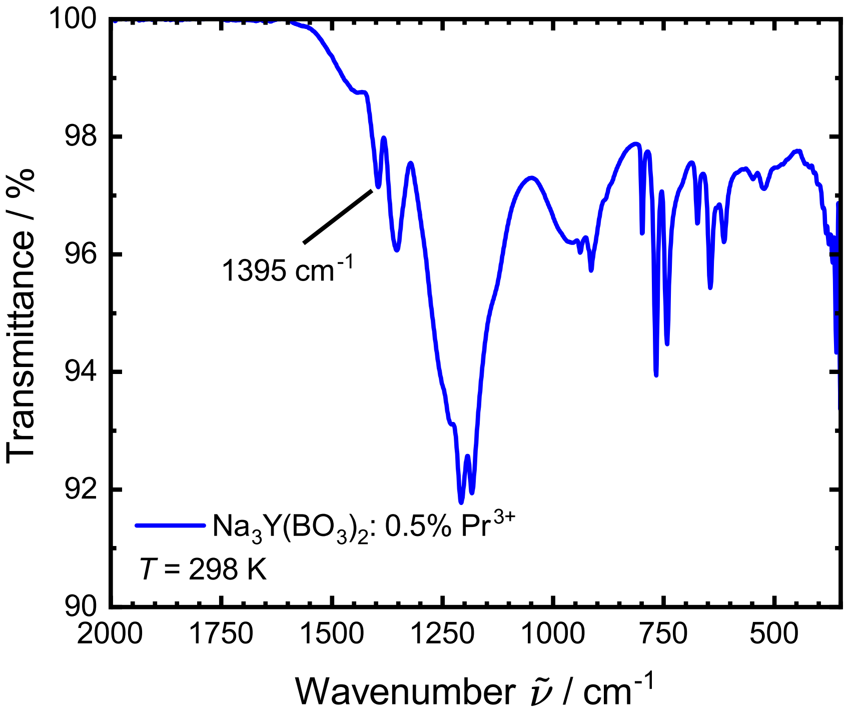

Figure S9: Infrared spectrum of the Pr^{3+}-activated $Na_3Y(BO_3)_2$. The cut-off phonon energy is given in the spectrum.

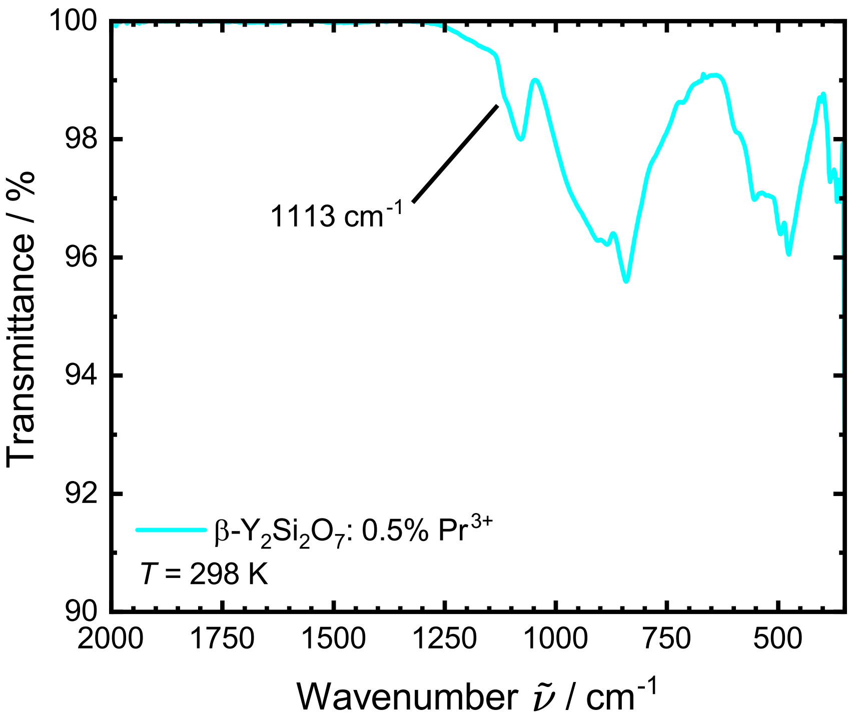

Figure S10: Infrared spectrum of the Pr^{3+}-activated β-$Y_2Si_2O_7$. The cut-off phonon energy is given in the spectrum.

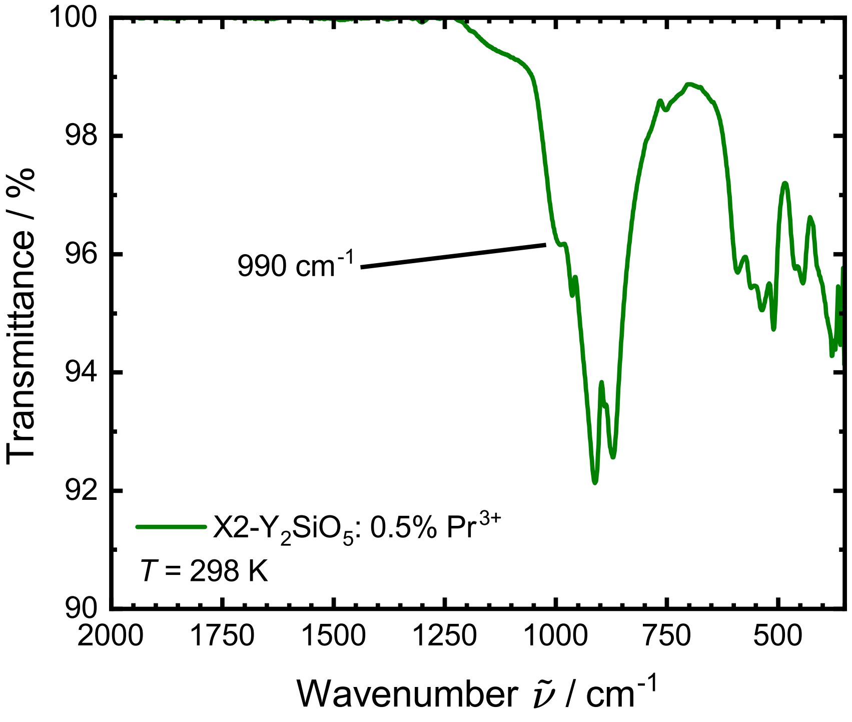

Figure S11: Infrared spectrum of the Pr^{3+}-activated X2-Y_2SiO_5. The cut-off phonon energy is given in the spectrum.

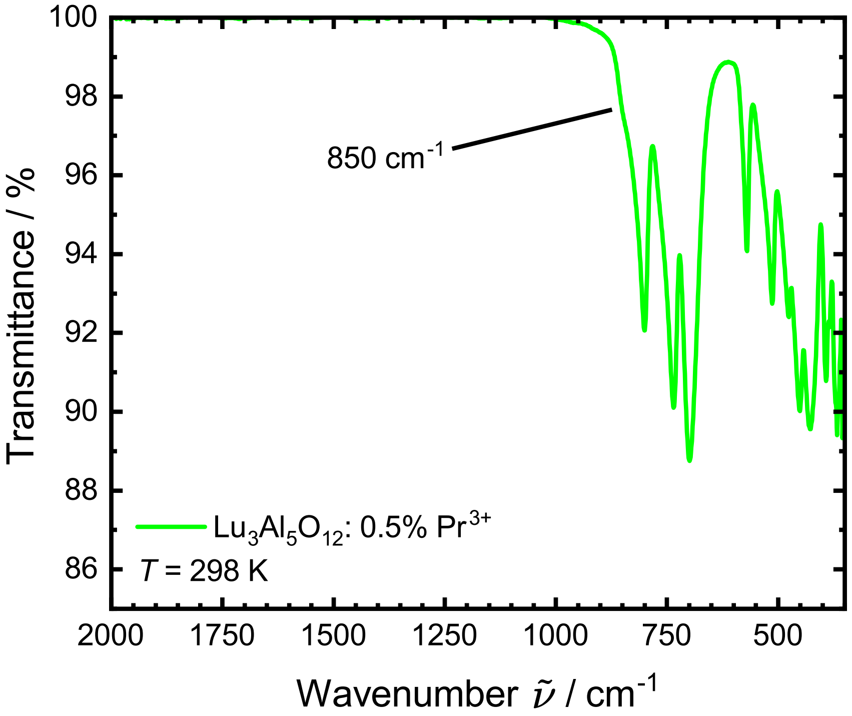

Figure S12: Infrared spectrum of the Pr^{3+}-activated $Lu_3Al_5O_{12}$. The cut-off phonon energy is given in the spectrum.

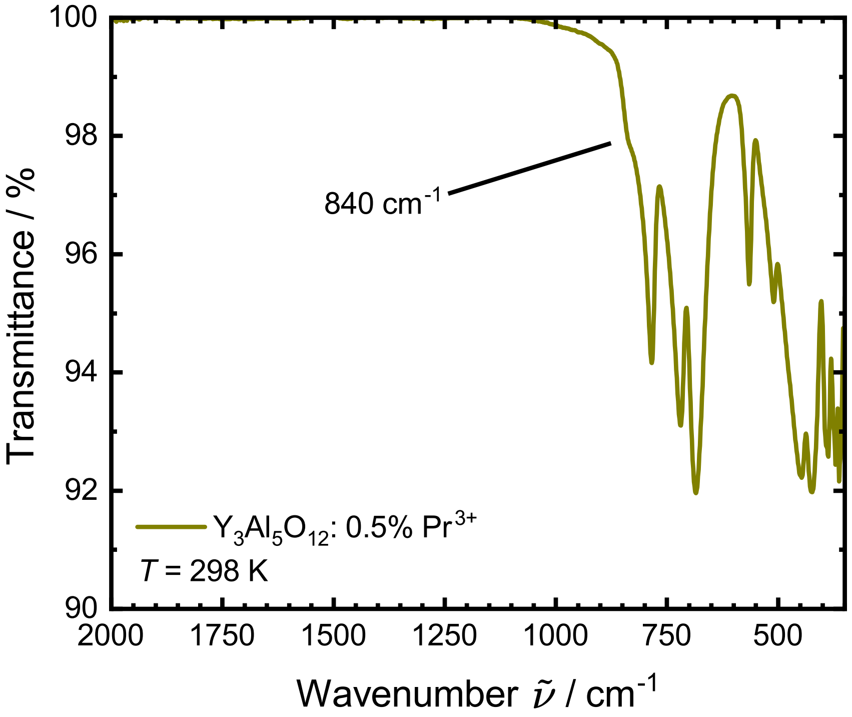

Figure S13: Infrared spectrum of the Pr^{3+}-activated $Y_3Al_5O_{12}$. The cut-off phonon energy is given in the spectrum.

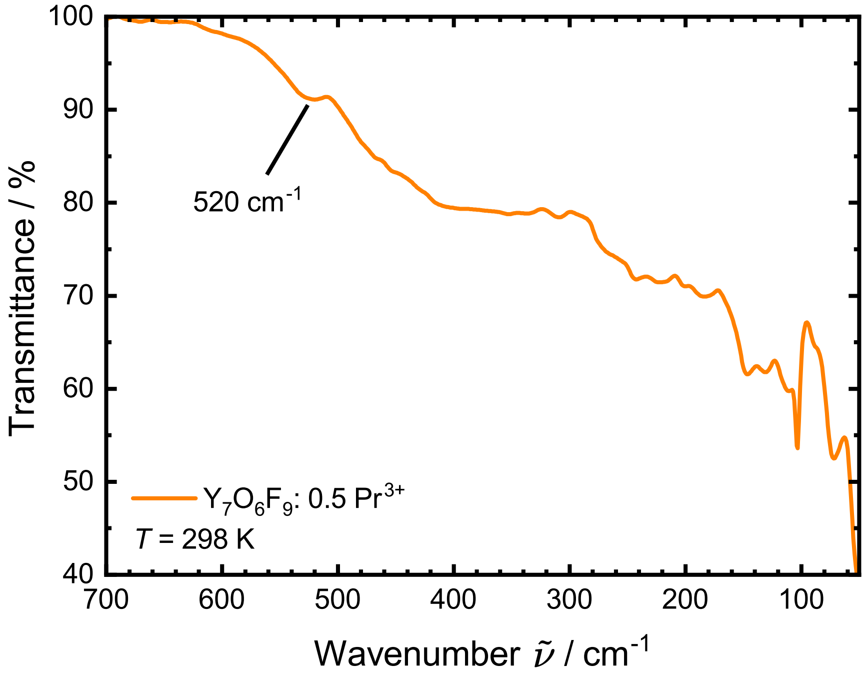

Figure S14: Infrared spectrum of the Pr^{3+}-activated $Y_7O_6F_9$. The cut-off phonon energy is given in the spectrum.

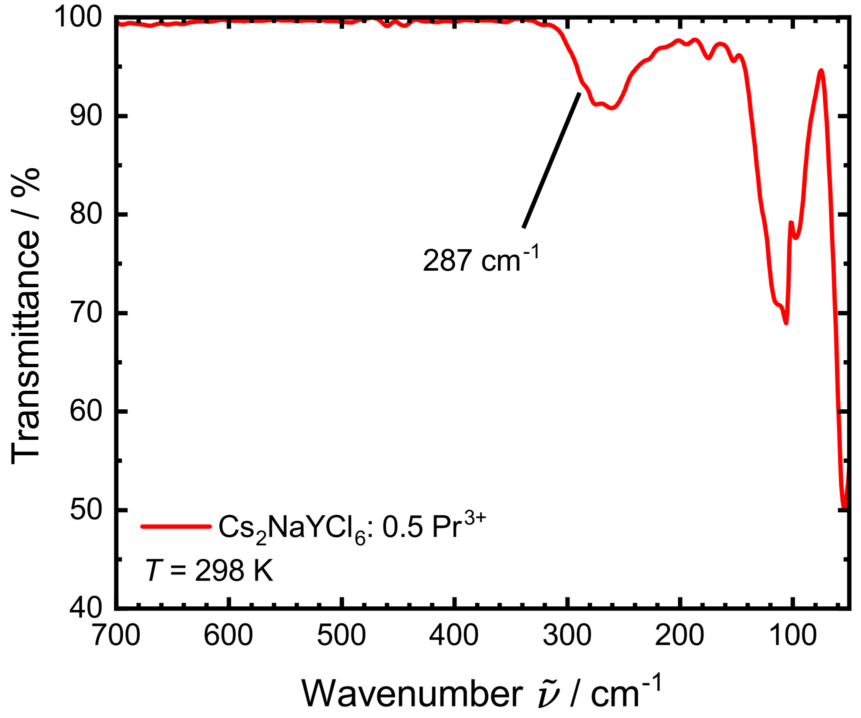

Figure S15: Infrared spectrum of the Pr^{3+}-activated Cs_2NaYCl_6. The cut-off phonon energy is given in the spectrum.

5 Upconversion luminescence

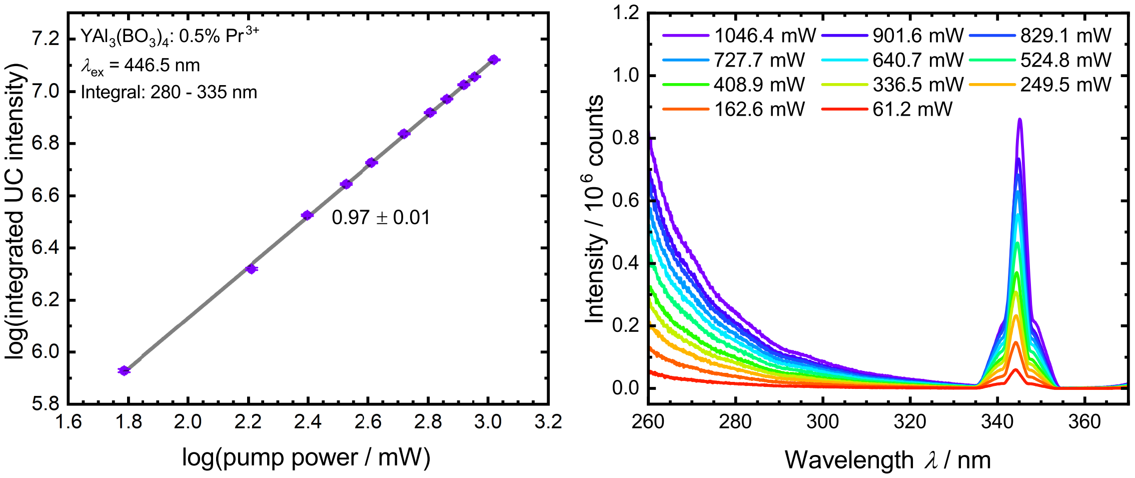

Figure S16: Left: Double logarithmic plot of integrated upconversion intensity of Pr^{3+}-activated $YAl_3(BO_3)_4$ against laser pump power with linear regression. The slope for the linear regression is given in the diagram. Right: UV upconversion spectra of Pr^{3+}-activated $YAl_3(BO_3)_4$ used for the double logarithmic plot. The slope of 1 results from the strong scattered light compared to the UC luminescence.

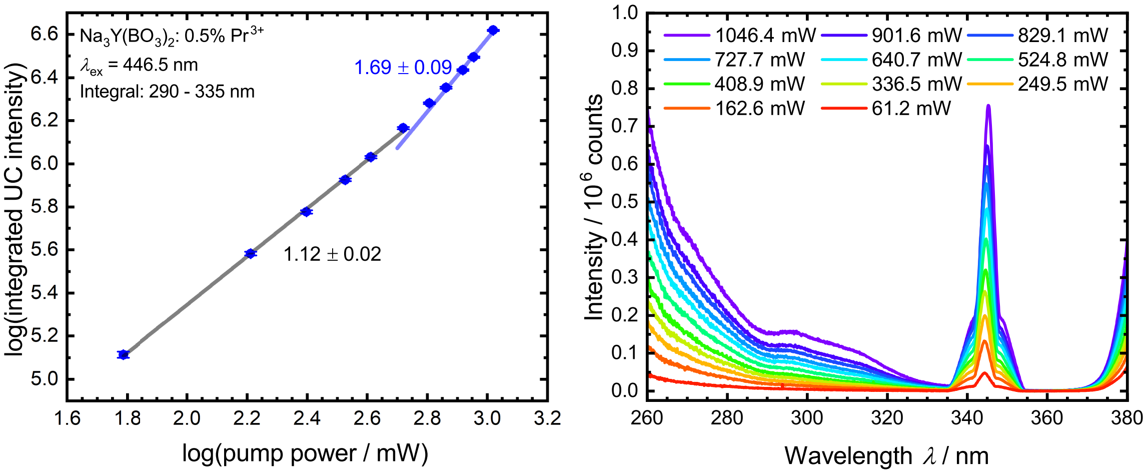

Figure S17: Left: Double logarithmic plot of integrated upconversion intensity of Pr^{3+}-activated $Na_3Y(BO_3)_2$ against laser pump power with linear regression. The slope for the linear regression is given in the diagram. Right: UV upconversion spectra of Pr^{3+}-activated $Na_3Y(BO_3)_2$ used for the double logarithmic plot. The increasing slope results from the interaction of scattered light (dominates at low pump power) and UC luminescence (dominates at higher pump power).

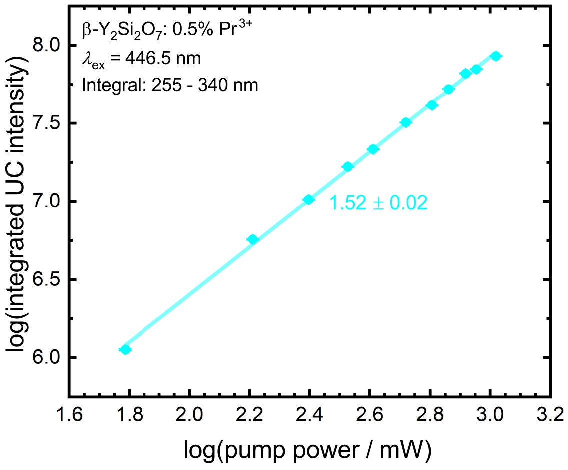

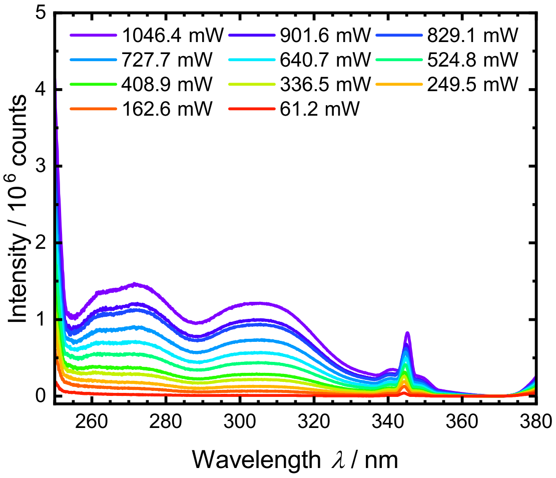

Figure S18: Left: Double logarithmic plot of integrated upconversion intensity of Pr^{3+}-activated β-$Y_2Si_2O_7$ against laser pump power with linear regression. The slope for the linear regression is given in the diagram. Right: UV upconversion spectra of Pr^{3+}-activated β-$Y_2Si_2O_7$ used for the double logarithmic plot.

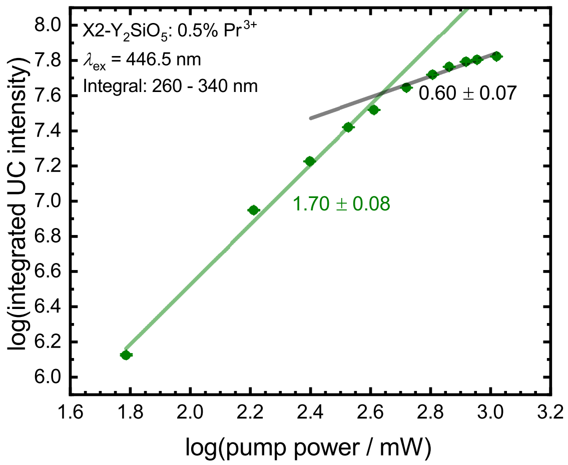

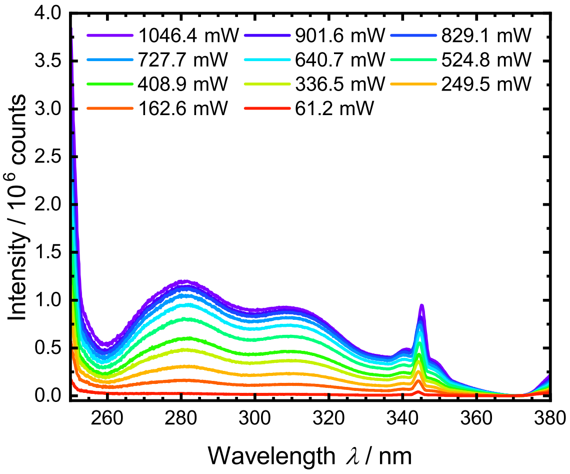

Figure S19: Left: Double logarithmic plot of integrated upconversion intensity of Pr^{3+}-activated X2-Y_2SiO_5 against laser pump power with linear regression. The slope for the linear regression is given in the diagram. Right: UV upconversion spectra of Pr^{3+}-activated X2-Y_2SiO_5 used for the double logarithmic plot.

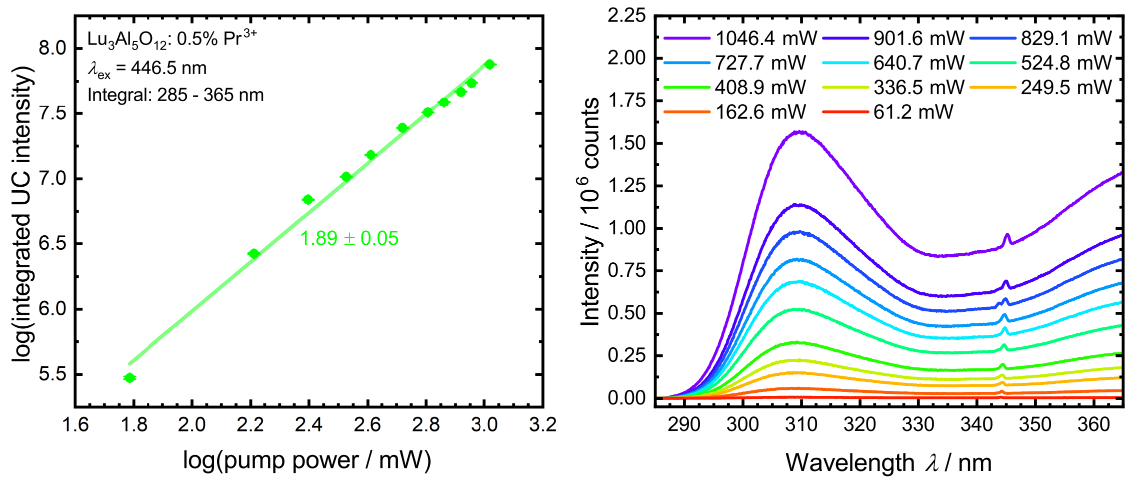

Figure S20: Left: Double logarithmic plot of integrated upconversion intensity of Pr^{3+}-activated $Lu_3Al_5O_{12}$ against laser pump power with linear regression. The slope for the linear regression is given in the diagram. Right: UV upconversion spectra of Pr^{3+}-activated $Lu_3Al_5O_{12}$ used for the double logarithmic plot.

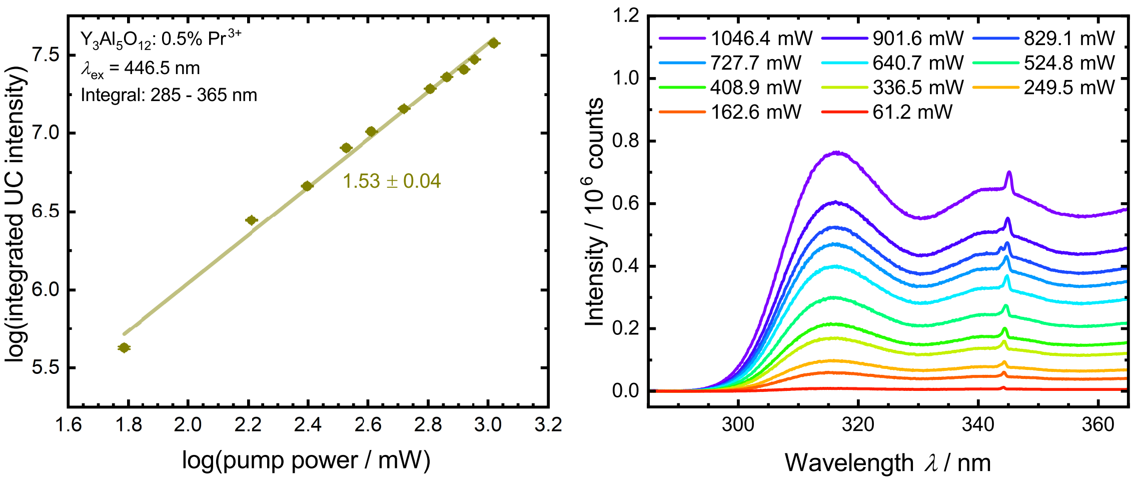

Figure S21: Left: Double logarithmic plot of integrated upconversion intensity of Pr^{3+}-activated $Y_3Al_5O_{12}$ against laser pump power with linear regression. The slope for the linear regression is given in the diagram. Right: UV upconversion spectra of Pr^{3+}-activated $Y_3Al_5O_{12}$ used for the double logarithmic plot.

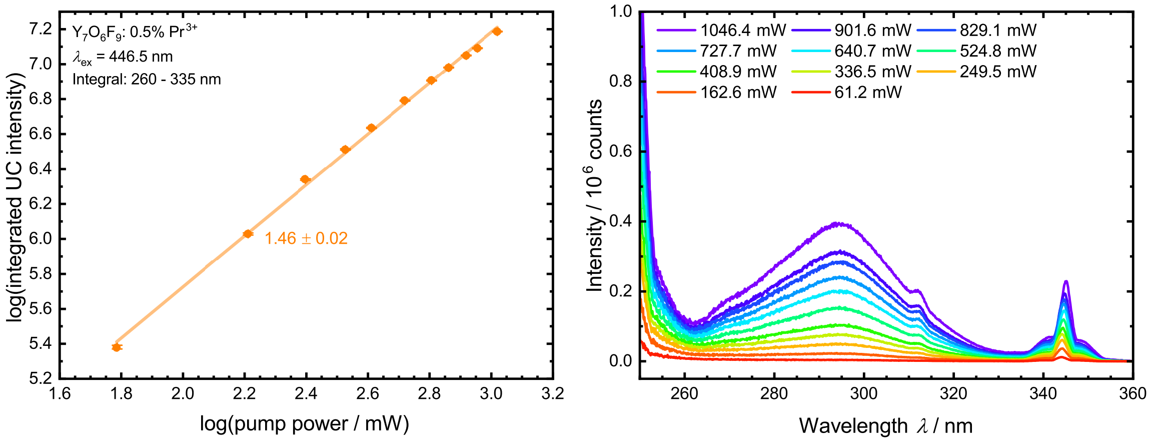

Figure S22: Left: Double logarithmic plot of integrated upconversion intensity of Pr^{3+}-activated $Y_7O_6F_9$ against laser pump power with linear regression. The slope for the linear regression is given in the diagram. Right: UV upconversion spectra of Pr^{3+}-activated $Y_7O_6F_9$ used for the double logarithmic plot.

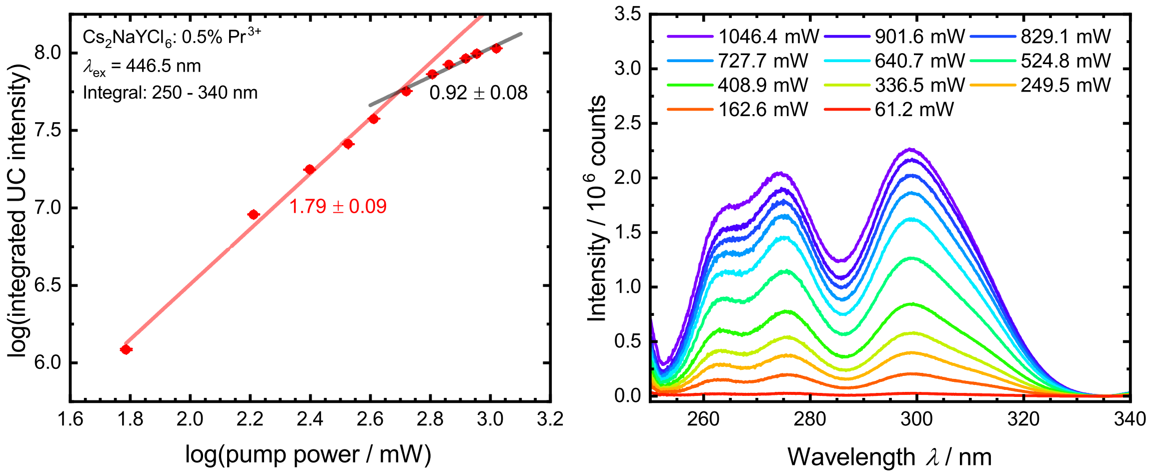

Figure S23: Left: Double logarithmic plot of integrated upconversion intensity of Pr^{3+}-activated Cs_2NaYCl_6 against laser pump power with linear regression. The slope for the linear regression is given in the diagram. Right: UV upconversion spectra of Pr^{3+}-activated Cs_2NaYCl_6 used for the double logarithmic plot.

6 Decay curves of $Cs_2NaY_{1\text{-}x}Pr_xCl_6$, (x = 0.005, 0.01)

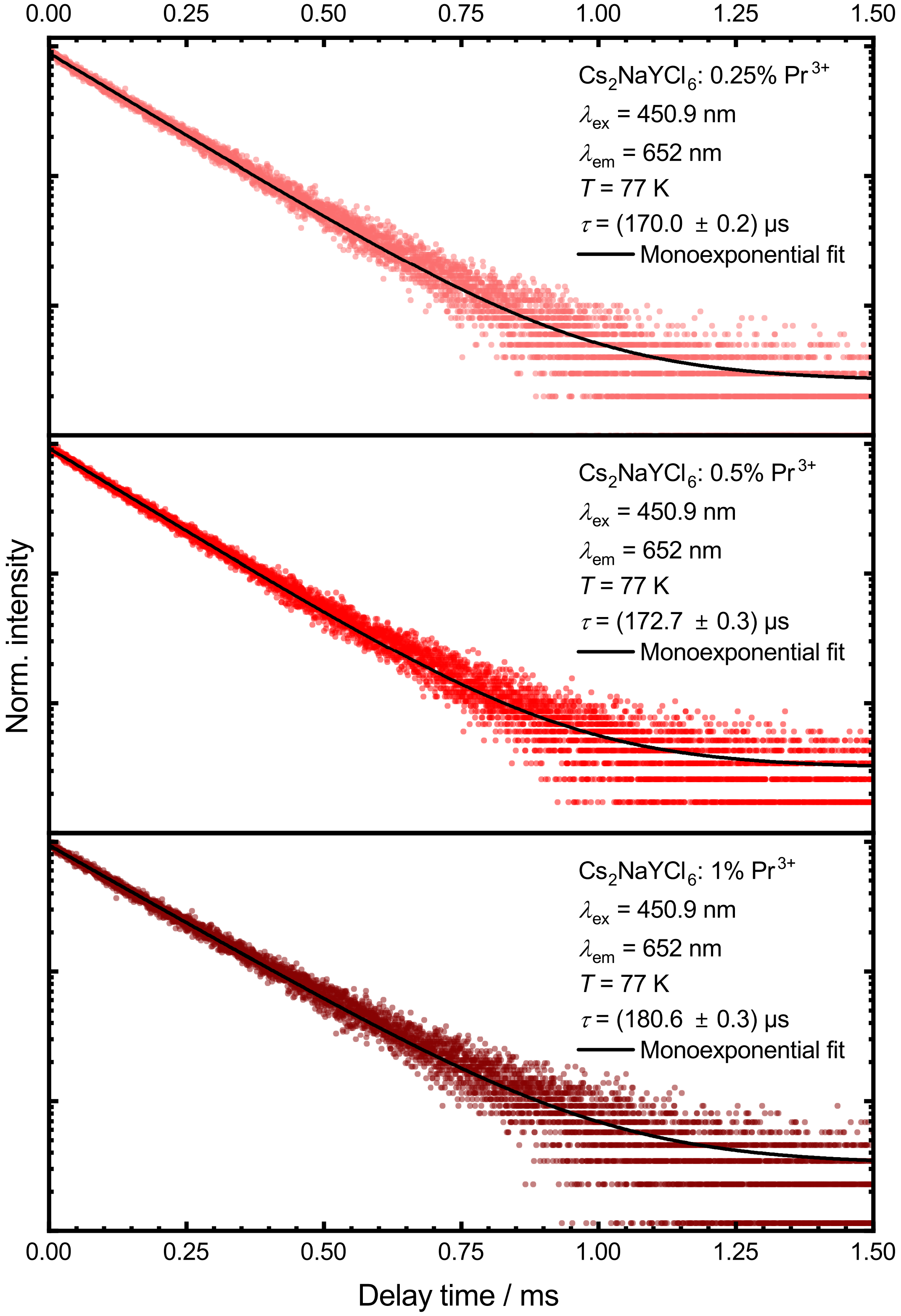

Figure S24: Photoluminescence decay curves of the $^3P_0$ level of $Cs_2NaY_{1\text{-}x}Pr_xCl_6$, (x = 0.0025, 0.005, 0.01) under 450.9 nm excitation ($^3P_2 \leftarrow {}^3H_4$) at 77 K. Monoexponential fits are given as black lines.

7 Rate equations of the upconversion mechanism

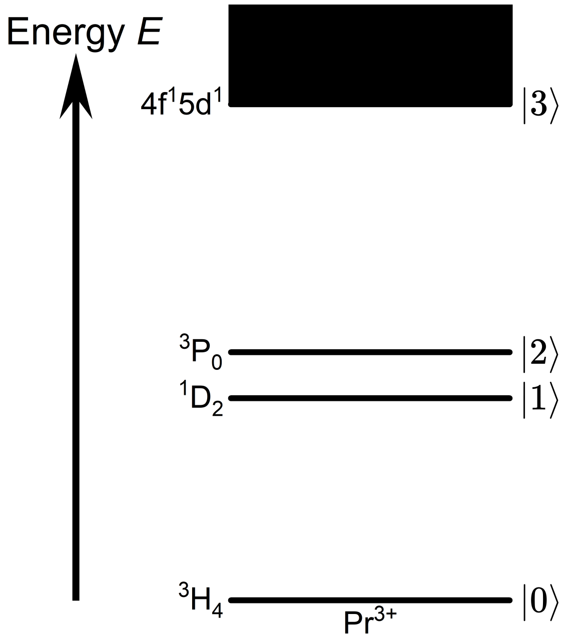

Figure S25: Simplified energy diagram of Pr^{3+} showing the involving states of the blue-to-UV upconversion process.

The rate equations for ESA mechanism involving a two sequential one-photon absorption step are given by

$$\frac{\mathrm{d}n_1}{\mathrm{d}t} = n_2 \cdot k_{2\mathrm{nr}}(T) - n_1 \cdot \frac{I\sigma_{13}}{\hbar\omega} - n_1 \cdot [k_{1\mathrm{r}} + k_{1\mathrm{nr}}(T)] \tag{3}$$

$$\frac{\mathrm{d}n_2}{\mathrm{d}t} = n_0 \cdot \frac{I\sigma_{02}}{\hbar\omega} - n_2 \cdot \frac{I\sigma_{23}}{\hbar\omega} - n_2 \cdot [k_{2\mathrm{r}} + k_{2\mathrm{nr}}(T)] \tag{4}$$

$$\frac{\mathrm{d}n_3}{\mathrm{d}t} = n_1 \cdot \frac{I\sigma_{13}}{\hbar\omega} + n_2 \cdot \frac{I\sigma_{23}}{\hbar\omega} - n_3 \cdot [k_{3\mathrm{r}} + k_{3\mathrm{nr}}(T)] \tag{5}$$

with subscripts 0, 1, 2, 3 refering to the $^3H_4 \equiv |0\rangle$ ground level, the $^1D_2 \equiv |1\rangle$ and the $^3P_0 \equiv |2\rangle$ levels as intermediate levels and the $4f^15d^1 \equiv |3\rangle$ configuration states, respectively (see Figure S25). The population densities n_i of state $|i\rangle$ depends on the pump light intensity I (in W cm^{-2}), the absorption cross-section from $|i\rangle$ to $|j\rangle$ level σ_{ij}, the pump photon energy $\hbar\omega$, the radiative decay rate $k_{i\mathrm{r}}$ of the corresponding level and the non-radiative decay rate $k_{i\mathrm{nr}}$. Under the assumption of steady-state conditions ($\frac{\mathrm{d}n_i}{\mathrm{d}t} = 0$), the rate Equations 3, 4 and 5 can be described as

$$n_2 \cdot k_{2\mathrm{nr}}(T) = n_1 \cdot [k_{1\mathrm{r}} + k_{1\mathrm{nr}}(T)] + n_1 \cdot \frac{I\sigma_{13}}{\hbar\omega} \tag{6}$$

$$n_0 \cdot \frac{I\sigma_{02}}{\hbar\omega} = n_2 \cdot \left\{\frac{I\sigma_{23}}{\hbar\omega} + [k_{2\mathrm{r}} + k_{2\mathrm{nr}}(T)]\right\} \tag{7}$$

$$n_3 \cdot [k_{3\mathrm{r}} + k_{3\mathrm{nr}}(T)] = n_2 \cdot \frac{I\sigma_{23}}{\hbar\omega} + n_1 \cdot \frac{I\sigma_{13}}{\hbar\omega} \tag{8}$$

According to that, the population of the $^1D_2$ level, the $^3P_0$ level and the $4f^15d^1$-derived states become

$$n_1 = \frac{n_0 \cdot \frac{I\sigma_{02}}{\hbar\omega} \cdot k_{2\mathrm{nr}}(T)}{\left\{\frac{I\sigma_{23}}{\hbar\omega} + [k_{2\mathrm{r}} + k_{2\mathrm{nr}}(T)]\right\} \cdot \left\{\frac{I\sigma_{13}}{\hbar\omega} + [k_{1\mathrm{r}} + k_{1\mathrm{nr}}(T)]\right\}} \tag{9}$$

$$n_2 = \frac{n_0 \cdot \frac{I\sigma_{02}}{\hbar\omega}}{\frac{I\sigma_{23}}{\hbar\omega} + [k_{2\mathrm{r}} + k_{2\mathrm{nr}}(T)]} \tag{10}$$

$$\begin{aligned} n_3 = & \frac{n_0 \frac{\sigma_{02}\sigma_{23}}{(\hbar\omega)^2} \cdot I^2}{\left\{\frac{I\sigma_{23}}{\hbar\omega} + [k_{2\mathrm{r}} + k_{2\mathrm{nr}}(T)]\right\} [k_{3\mathrm{r}} + k_{3\mathrm{nr}}(T)]} \\ & + \frac{n_0 \frac{\sigma_{02}\sigma_{13}}{(\hbar\omega)^2} \cdot I^2}{\left\{\frac{I\sigma_{23}}{\hbar\omega} + [k_{2\mathrm{r}} + k_{2\mathrm{nr}}(T)]\right\} \left\{\frac{I\sigma_{13}}{\hbar\omega} + [k_{1\mathrm{r}} + k_{1\mathrm{nr}}(T)]\right\} [k_{3\mathrm{r}} + k_{3\mathrm{nr}}(T)]} \end{aligned} \tag{11}$$

A further consideration of Equations 9, 10 and 11 results in two limiting cases for the populations depending on the intensity:

For a fast decay of the intermediate levels or a low pump light intensity $\left([k_{i\mathrm{r}} + k_{i\mathrm{nr}}(T)] \gg \frac{I\sigma_{i3}}{\hbar\omega}; i = 1, 2\right)$, the population of the $^3P_0$ and the $^1D_2$ level are proportional to the intensity ($n_{1,2} \propto I$), while the population of the $4f^15d^1$ configuration states is proportional to the square of the intensity ($n_3 \propto I^2$) for a two-photon process.

For a slow decay of the intermediate levels or a high pump light intensity $\left([k_{i\mathrm{r}} + k_{i\mathrm{nr}}(T)] \ll \frac{I\sigma_{i3}}{\hbar\omega}\right)$, the population of the $^1D_2$ level is proportional to I^{-1} and the population of the $^3P_0$ level levels off and becomes constant ($n_2 \propto$ const.), while the population of the $4f^15d^1$ configuration states is proportional to the intensity ($n_3 \propto I$).

For the ETU mechanism, the rate equations are given by

$$\frac{\mathrm{d}n_1}{\mathrm{d}t} = k_{2\mathrm{nr}}(T) \cdot n_2 - [k_{1\mathrm{r}} + k_{1\mathrm{nr}}(T)] \cdot n_1 - [w_{12} + w_{21}] \cdot n_1 \cdot n_2 \tag{12}$$

$$\frac{\mathrm{d}n_2}{\mathrm{d}t} = n_0 \cdot \frac{I\sigma_{02}}{\hbar\omega} - [k_{2\mathrm{r}} + k_{2\mathrm{nr}}(T)] \cdot n_2 - 2w_{22} \cdot n_2^2 - [w_{12} + w_{21}] \cdot n_1 \cdot n_2 \tag{13}$$

$$\frac{\mathrm{d}n_3}{\mathrm{d}t} = w_{22} \cdot n_2^2 + [w_{12} + w_{21}] \cdot n_1 \cdot n_2 - [k_{3\mathrm{r}} + k_{3\mathrm{nr}}(T)] \cdot n_3 \tag{14}$$

with the upconversion rate w_{ij} between the two states $|i\rangle$ and $|j\rangle$. It is assumed that there is no upconversion starting from two Pr^{3+} ions in the $^1D_2$ level ($w_{11} = 0$), as the resonance condition is not fulfilled here for most compounds (**??**). Under steady-state conditions, the rate equations can be described as

$$k_{2\mathrm{nr}}(T) \cdot n_2 = [k_{1\mathrm{r}} + k_{1\mathrm{nr}}(T)] \cdot n_1 + [w_{12} + w_{21}] \cdot n_1 \cdot n_2 \tag{15}$$

$$n_0 \cdot \frac{I\sigma_{02}}{\hbar\omega} = [k_{2\mathrm{r}} + k_{2\mathrm{nr}}(T)] \cdot n_2 + 2w_{22} \cdot n_2^2 + [w_{12} + w_{21}] \cdot n_1 \cdot n_2 \tag{16}$$

$$[k_{3\mathrm{r}} + k_{3\mathrm{nr}}(T)] \cdot n_3 = w_{22} \cdot n_2^2 + [w_{12} + w_{21}] \cdot n_1 \cdot n_2 \tag{17}$$

For the ETU mechanism, again, two limiting cases can occur:

For a fast decay of the intermediate levels or a low upconversion rate

($w_{ij} \ll [k_{i\mathrm{r}} + k_{i\mathrm{nr}}(T)]$), Equations 15 and 16 can be written as

$$k_{2\mathrm{nr}}(T) \cdot n_2 \approx [k_{1\mathrm{r}} + k_{1\mathrm{nr}}(T)] \cdot n_1 \tag{18}$$

$$n_0 \cdot \frac{I\sigma_{02}}{\hbar\omega} \approx [k_{2\mathrm{r}} + k_{2\mathrm{nr}}(T)] \cdot n_2 \tag{19}$$

which reveals that the population of the $^3\mathrm{P}_0$ and the $^1\mathrm{D}_2$ levels are proportional to the pump light intensity ($n_{1,2} \propto I$). For the population of the $4\mathrm{f}^1 5\mathrm{d}^1$-derived states, Equation 17 then results in a quadratic dependence on the intensity ($n_3 \propto I^2$), in complete analogy to the case of the ESA mechanism.

For a slow decay of the intermediate levels or a high upconversion rate ($w_{ij} \gg [k_{i\mathrm{r}} + k_{i\mathrm{nr}}(T)]$), Equations 15 and 16 can be written as

$$k_{2\mathrm{nr}}(T) \cdot n_2 \approx [w_{12} + w_{21}] \cdot n_1 \cdot n_2 \tag{20}$$

$$n_0 \cdot \frac{I\sigma_{02}}{\hbar\omega} \approx 2w_{22} \cdot n_2^2 + [w_{12} + w_{21}] \cdot n_1 \cdot n_2 \tag{21}$$

which shows the population of the $^1\mathrm{D}_2$ level is independent of the irradiated intensity and becomes constant ($n_1 \propto$ const.), while the population of the $^3\mathrm{P}_0$ level is proportional to the square root of the pump light intensity ($n_2 \propto I^{\frac{1}{2}}$). For the population of the $4\mathrm{f}^1 5\mathrm{d}^1$-derived states, Equation 17 then results in a linear dependence on the intensity ($n_3 \propto I$).

References

[1] J. Collins, M. Geen, M. Bettinelli, B. Di Bartolo, *Journal of Luminescence* **2012**, *132*, 2626–2633, DOI https://doi.org/10.1016/j.jlumin.2012.04.027.

[2] S. Tian, H. Wu, Y. Fu, L. Huai, W. Wang, Z. Ma, J. Zhang, H. Li, *Ceram. Int.* **2018**, *44*, 23293–23296, DOI https://doi.org/10.1016/j.ceramint.2018.09.037.

[3] V. Naresh, B. S. Ham, *Journal of Alloys and Compounds* **2016**, *664*, 321–330, DOI https://doi.org/10.1016/j.jallcom.2015.12.246.

[4] D. Yu, T. Yu, A. J. van Bunningen, Q. Zhang, A. Meijerink, F. T. Rabouw, *Light Sci. Appl.* **2020**, *9*, DOI https://doi.org/10.1038/s41377-020-00346-z.

[5] D. Yu, H. Li, D. Zhang, Q. Zhang, A. Meijerink, M. Suta, *Light Sci. Appl.* **2021**, *10*, DOI https://doi.org/10.1038/s41377-021-00677-5.

[6] Q. Shi, J. Cui, F. You, S. Huang, H. Peng, Y. Huang, *J. Lumin.* **2019**, *213*, 489–493, DOI https://doi.org/10.1016/j.jlumin.2019.05.037.

[7] H. Wang, L. Gao, K. Niihara, *Materials Science and Engineering: A* **2000**, *288*, 1–4, DOI https://doi.org/10.1016/S0921-5093(00)00904-7.

[8] L. R. Morss, M. Siegal, L. Stenger, N. Edelstein, *Inorg. Chem.* **1970**, *9*, 1771–1775.

[9] Z. Hasan, L. Biyikli, M. J. Sellars, G. A. Khodaparast, F. S. Richardson, J. R. Quagliano, *Phys. Rev. B* **1997**, *56*, 4518–4528, DOI https://doi.org/10.1103/PhysRevB.56.4518.

[10] A. A. Coelho, *Journal of Applied Crystallography* **2018**, *51*, 210–218.

[11] F. Schröder, P. Pues, D. Enseling, T. Jüstel, *Luminescence* **2023**, *38*, 702–708, DOI https://doi.org/10.1002/bio.4496.

[12] E. Belokoneva, A. Azizov, N. Leonyuk, M. Simonov, N. Belov, *J. Struct. Chem+.+* **1981**, *22*, 476–478.

[13] Y Zhang, X. Chen, J. Liang, T Xu, *J. Alloy. Compd.* **2002**, *333*, 72–75, DOI https://doi.org/10.1016/S0925-8388(01)01689-9.

[14] G. J. Redhammer, G. Roth, *Acta Crystallographica Section C Crystal Structure Communications* **2003**, *59*, i103–i106, DOI http://dx.doi.org/10.1107/S0108270103018869.

[15] B. Maksimov, V. Ilyukhin, Y. A. Kharitonov, N. Belov, *Kristallografiya+.+* **1970**, *15*, 926–932.

[16] L. Ding, Q. Zhang, J. Luo, W. Liu, W. Zhou, S. Yin, *J. Alloy. Compd.* **2011**, *509*, 10167–10171, DOI http://dx.doi.org/10.1016/j.jallcom.2011.07.083.

[17] L. Dobrzycki, E. Bulska, D. A. Pawlak, Z. Frukacz, K. Woźniak, *Inorg. Chem.* **2004**, *43*, 7656–7664, DOI http://doi.org/10.1021/ic049920z.

[18] D. J. M. Bevan, A. W. Mann, *Acta Crystallographica Section B Structural Crystallography and Crystal Chemistry* **1975**, *31*, 1406–1411, DOI 10.1107/S0567740875005298.

[19] R. Sabry-Grant, M. Vickers, J. K. Cockcroft, *Z. für Krist.* **2007**, *222*, 356–364, DOI http://doi.org/10.1524/zkri.2007.222.7.356.

[20] J. Mooney, P. Kambhampati, *The Journal of Physical Chemistry Letters* **2013**, *4*, 3316–3318, DOI https://doi.org/10.1021/jz401508t.
